# Continuous spectral and coupling-strength encoding with dual-gradient metasurfaces

**DOI:** 10.1038/s41565-024-01767-2

**Published:** 2024-08-26

**Authors:** Andreas Aigner, Thomas Weber, Alwin Wester, Stefan A. Maier, Andreas Tittl

**Affiliations:** 1 Chair in Hybrid Nanosystems, Nano-Institute Munich, Faculty of Physics, Ludwig-Maximilians-Universtität München, Munich, Germany; 2 School of Physics and Astronomy, Monash University, Clayton, Victoria Australia; 3 The Blackett Laboratory, Department of Physics, Imperial College London, London, UK

**Keywords:** Nanophotonics and plasmonics, Metamaterials, Nanocavities

## Abstract

To control and enhance light–matter interactions at the nanoscale, two parameters are central: the spectral overlap between an optical cavity mode and the material’s spectral features (for example, excitonic or molecular absorption lines), and the quality factor of the cavity. Controlling both parameters simultaneously would enable the investigation of systems with complex spectral features, such as multicomponent molecular mixtures or heterogeneous solid-state materials. So far, it has been possible only to sample a limited set of data points within this two-dimensional parameter space. Here we introduce a nanophotonic approach that can simultaneously and continuously encode the spectral and quality-factor parameter space within a compact spatial area. We use a dual-gradient metasurface design composed of a two-dimensional array of smoothly varying subwavelength nanoresonators, each supporting a unique mode based on symmetry-protected bound states in the continuum. This results in 27,500 distinct modes and a mode density approaching the theoretical upper limit for metasurfaces. By applying our platform to surface-enhanced molecular spectroscopy, we find that the optimal quality factor for maximum sensitivity depends on the amount of analyte, enabling effective molecular detection regardless of analyte concentration within a single dual-gradient metasurface. Our design provides a method to analyse the complete spectral and coupling-strength parameter space of complex material systems for applications such as photocatalysis, chemical sensing and entangled photon generation.

## Abstract (web summary)

A dual-gradient metasurface is introduced that allows continuous control over light–matter coupling in the spectral and quality-factor parameter space. Used for molecular sensing, it achieves mode densities near the theoretical limit for metasurfaces.

## Main

Optical cavities have substantially advanced our ability to manipulate light–matter interactions, with various applications ranging from lasers and spectroscopic techniques to quantum information processing^1^. Especially in nanoscience, nanoresonators^2,3^—the nanoscale counterparts of optical cavities—have bridged the size gap to materials like quantum dots, van der Waals materials and molecules. This allowed breakthroughs in photocatalysis^4–6^, entangled photon sources^7,8^, biochemical sensing^9,10^ and the study of polaritons^11,12^. The interaction of light and matter in nanoresonators is fundamentally governed by two key parameters: the spectral overlap of the optical mode with the excitation of the target system (for example, excitonic^13,14^ or molecular absorption lines^15,16^) and the strength of the interaction set by the resonators’ quality (*Q*) factor (defined as the resonance frequency divided by the line-width).

In terms of spectral overlap, the fundamental goal is to simultaneously amplify and probe the material’s dispersive properties. To achieve the necessary spectral coverage, research in multiresonant nanophotonic platforms has led to concepts like plasmonic oligomers^17,18^, multiresonant plasmonic surface lattice resonances^19,20^, dual-band perfect absorbers^21^, fractal plasmonics^22,23^ and non-local metasurfaces^24^. However, these platforms often face restrictions owing to the limited number of resonances that a single nanoresonator, or two-dimensional (2D) arrays of such resonators known as metasurfaces^25,26^, can support. While active metasurfaces allow some degree of tunability, they typically cannot cover wide spectral ranges^12,27^. Thus, researchers have turned to using metasurfaces with spatially varying resonator geometries that allow local adjustment of the optical response. Initially developed for non-resonant phase gradient metasurfaces^28,29^, this concept has been extended to resonant systems^30,31^.

Whereas the advancements for spectral coverage have been substantial, precisely tuning the resonators’ coupling strength remains challenging. Symmetry-protected bound states in the continuum (BICs) have recently been shown to be a promising approach^32,33^. By adjusting geometric parameters, BICs can fine-tune their resonance line-widths, controlling the strength of the light–matter interaction^34,35^. This has led to multispectral BIC-driven metasurfaces with up to 100 resonances on a single sensor platform^36,37^. While BICs are effective at probing the spectral and coupling space, they require extended arrays of identical resonators as BICs inherently are collective modes^38^. Each metasurface, consisting of at least hundreds of identical resonators, can probe only a single point in the 2D spectral–coupling parameter space. This prohibits the complete analysis of complex systems like multicomponent molecular mixtures that need extensive data to uncover the numerous interactions and effects occurring within them. Furthermore, it hinders the integration into hyperspectral optical systems and compact devices. Despite recent successes in creating spectrally tuned plasmonic gradients for refractive index sensing^39^ and dielectric gradients for higher harmonic generation^40^, a comprehensive platform for studying both the coupling space and the combined 2D spectral–coupling space remains elusive.

Here we introduce the concept of dual-gradient metasurfaces, seamlessly spanning the 2D parameter space of resonance wavelength and coupling strength, ideal for probing diverse light–matter coupling phenomena. First we explore spectral gradients that offer continuously spectrally tunable resonances. Experimentally, we investigate the effect of the gradients’ spectral coverage on the resonance performance. Strikingly, we find that for moderate widths, the performance of the gradient is equal to established monospectral metasurfaces, despite the perturbed periodicity. Similarly, we present the idea of coupling (*Q*-factor) gradients. Distinct from the widely used phase gradients that manipulate beam profiles in the far field^41–43^, our coupling gradients enable spatial mapping of light–matter interaction strength in the near field. Combining these advancements, we experimentally realize a dual-gradient metasurface with independently adjustable gradients, showcasing extensive and simultaneous spectral and coupling-strength coverage in a compact footprint. To demonstrate the dual gradient’s capabilities, we apply it to surface-enhanced molecular sensing, where a wide spectral coverage is needed to retrieve the unique vibrational fingerprints of molecules. We not only capture the spectral fingerprint but also unveil an additional coupling-based dimension of spectroscopic data, showcasing a concentration-based dependence between the resonances’ *Q*-factor and the detection sensitivity. This crucial insight was largely unnoticed as sampling of the coupling parameter space had been constrained to discrete points within it. Our dual gradient ensures optimal sensitivity across all sensing conditions, irrespective of the analyte concentration or the solvent used.

## Principle of dual-gradient metasurfaces

**Fig. 1:**
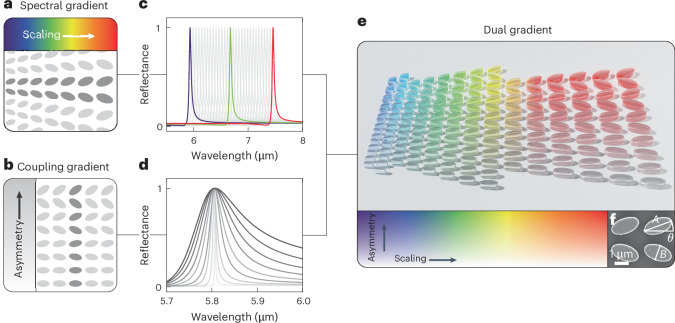
Concept of dual-gradient metasurfaces combining independent spectral and coupling gradients. **a**, Schematic representation of a spectral-gradient metasurface composed of unit cells of tilted ellipse pairs. Identical resonators form chains along the vertical, with a gradual increase in scaling along the horizontal. **b**, Schematic representation of a coupling-gradient metasurface. The asymmetry (in the form of the tilting angle *θ*) varies along the vertical, altering the far-field coupling strength and the resonance *Q*-factor. **c**, Numerical reflectance spectra of BIC resonances with scaling factors ranging from 1.0 to 1.3, increasing incrementally by steps of 0.01. **d**, Numerical reflectance spectra with a constant scaling factor (*S* = 1), and *θ* varying from 0° (light grey) to 45° (black) in increments of 5°. Spectral alignment of the resonances is achieved by using an additional scaling factor, discussed in Fig. 3. **e**, Illustration of a dual-gradient metasurface that incorporates both a spectral gradient along the horizontal and a coupling gradient along the vertical, illustrated by a spectral gradient along the *x* axis and a saturation gradient along the *y* axis, respectively. **f**, SEM image of the final metasurfaces showing two unit cells. *A* and *B* represent the long and short axes of the ellipses, and *θ* is the tilt angle.

We chose a BIC-driven metasurface geometry consisting of pairs of tilted amorphous silicon ellipses on top of an infrared-transparent calcium fluoride (CaF_2_) substrate (Supplementary Fig. 1a for a detailed sketch of the unit cell and relevant dimensions). This particular unit cell design is ideal for our target molecular spectroscopy application due to its strong surface-confined electromagnetic fields, low baseline reflectance and fabrication robustness^44^. Extending the traditional approach of arranging the elliptical resonators in a 2D periodic array, we introduce a multiplicative lateral scaling factor *S* (Supplementary Fig. 2) to modify the dimensions of each unit cell, creating spectral gradients (Fig. 1a). Spectral resonance tuning is achieved by continuously varying *S*, creating an unambiguous mapping between spatial and spectral information. The photonic behaviour of symmetry-protected BIC metasurfaces is fundamentally determined by the asymmetry factor *α*, defined as the sine of the ellipse tilt angle *θ*, that is, *α* = sin(*θ*) (ref. ^45^). The asymmetry controls the radiative coupling of the resonance to the far field and therefore governs the resonance’s *Q*-factor, the ratio of the resonance frequency to the resonance line-width (Supplementary Fig. 1b,c). By arranging unit cells with identical *S* but varying *α* into a 2D lattice, we generate a coupling gradient that provides a broad range of *Q*-factors within a single metasurface (Fig. 1b).

Following extensive numerical optimization, targeted at achieving BICs on the long wavelength side of the Rayleigh limit^46^, even for high asymmetries *θ* of up to 45° (Supplementary Fig. 1d–i for details), we have selected a unit cell geometry with a pitch of *P*_*x*_ = 2.4 μm in the *x* direction, a pitch of *P*_*y*_ = 4 μm in the *y* direction, an ellipse long diameter *A* of 2 μm, a short diameter *B* of 1 μm and a height *h* of 0.75 μm. The numerical results reveal that adjusting the scaling factor from 1.0 to 1.3 along the *x* axis for *θ* = 20° shifts the pronounced reflectance peak of the BIC resonance from 5.9 μm to 7.2 μm, underlining the distinct spatially dependent response of a spectral-gradient metasurface (Fig. 1c). Similarly, Fig. 1d presents numerical results for a fixed scaling factor (*S* = 1) with the tilting angle *θ* ranging from 0 to 45°, generating the spatially dependent response of a coupling gradient. We observe a broadening of the mode with increasing angles, following the typical *Q*-factor relationship for symmetry-protected BICs, in this case *Q* = 1/sin^2^(*θ*). The simultaneous parameter variation of the dual gradient is created by merging both resonance tuning mechanisms (Fig. 1e), allowing the metasurface to seamlessly encode a wide range of spectral and coupling-strength information.

## Spectral gradients

**Fig. 2:**
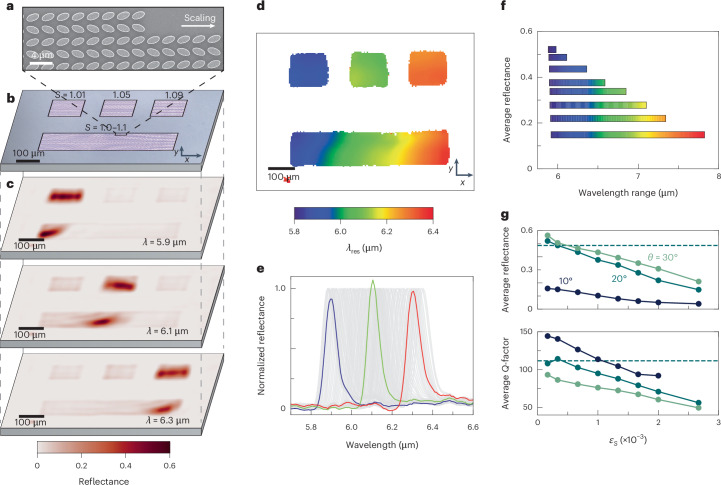
Spectral-gradient metasurface. **a**, SEM image of a spectral gradient with the scaling along the *x* axis. **b**, Optical image of the three monospectral metasurfaces with scaling factors *S* = 1.01, 1.05 and 1.09 from left to right, and the spectral gradient with *S* = 1.0–1.1 (below). **c**, Reflectance snapshots of the metasurfaces shown in **b**, taken at wavelengths (*λ*) of 5.9, 6.1 and 6.3 µm. **d**, Colour-coded resonance wavelengths *λ*_res_ for each detector pixel. Non-resonant pixels are shown in white. **e**, Normalized reflectance spectra taken from the spectral gradient along the *x* axis, shown in grey. The coloured spectra correspond to the three monospectral metasurfaces, each normalized to the gradient’s maximum reflectance at their respective spectral position. A direct reflectance comparison of the monospectral metasurfaces with the gradient at the equivalent scaling position is shown in Supplementary Fig. 9. **f**, Average reflectance amplitude and wavelength range for gradients with *θ* = 20° of different gradient steepness. **g**, Average reflectance amplitude and *Q*-factor plotted against different scaling increments *ε*_*S*_ for gradients with *θ* = 10, 20 and 30°. The horizontal dashed line represents the values for the monospectral metasurfaces with *θ* = 20°.

We initiate our experimental demonstration of gradient metasurfaces by investigating the performance of continuous spectral gradients with a fixed tilting angle *θ* = 20° and varying lateral scaling *S*. Using high-resolution electron-beam lithography and reactive ion etching, a spectral gradient with continuous unit cell scaling along the *x* axis from *S* = 1.0 to 1.1 over a length of 600 µm was realized (scanning electron microscopy (SEM) image of two unit cells in Fig. 1f, a section of the spectral gradient in Fig. 2a and an angled view in Supplementary Fig. 3). In our study, we fabricate monospectral metasurfaces each measuring 150 × 150 µm^2^ and consisting of 35 × 59 unit cells. These dimensions are sufficient to support symmetry-protected BICs^36,47,48^ and are further confirmed by an experimental monospectral metasurfaces size sweep in Supplementary Fig. 4. The metasurfaces, with scaling factors *S* = 1.01, 1.05 and 1.09, were fabricated close to the spectral gradient for a direct comparison (optical image in Fig. 2b). The monospectral metasurfaces were chosen so that they correspond to points in the centre and close to the ends of the spectral gradient, with resonance wavelengths of 5.9 µm, 6.1 µm and 6.3 µm. The metasurfaces were optically characterized using a multispectral imaging microscope incorporating tunable quantum cascade lasers and a 480 pixel × 480 pixel imaging detector (Supplementary Fig. 5a), allowing us to record snapshots of the reflectance signal at different wavelengths.

At each of the three wavelengths given above, one of the monospectral metasurfaces exhibits a high reflectance amplitude, indicating resonant nanostructures. The spectral gradient, however, shows pronounced reflectance zones (cross-sections for 6.1 µm in Supplementary Fig. 6) across all three wavelengths, demonstrating resonant behaviour throughout (Fig. 2c). Supplementary Video 1 illustrates this unique resonant behaviour, where each frame represents the reflectance response for a specific wavelength. In striking contrast to the monospectral metasurfaces, the peak reflectance map across all wavelengths shows a consistently high reflectance signal over the whole spectral gradient, indicating efficient and continuous resonance coverage (Supplementary Fig. 5b and rotated polarization in Supplementary Fig. 7a). This advantageous behaviour is further highlighted by plotting the extracted resonance wavelengths *λ*_res_ for each image pixel (Fig. 2d), where the spectral gradient reveals a smooth transition from 5.9 to 6.4 µm, compared to the solid colours associated with the discrete metasurface pixels. The lower reflectance amplitude in the upper portion of both the gradient and the monospectral metasurfaces can be attributed to a slight angle of the illuminating laser beam (Supplementary Fig. 8).

To further assess the performance of the spectral gradient, we extract normalized reflectance spectra from equally distributed points along the gradient’s *x* axis (Fig. 2e, grey lines) and compare them to the monospectral metasurfaces, normalized to the reflectance amplitudes of the gradient at the corresponding resonance positions (Fig. 2e, coloured lines). Strikingly, the spectra taken from the gradient with Δ*S* = 0.1 show nearly identical reflectance amplitudes compared to the monospectral metasurfaces (Supplementary Fig. 9). Furthermore, we observe good agreement between simulations and experiments, especially when introducing parasitic losses into the simulations (Supplementary Note 1 and Supplementary Fig. 10).

As a next step, we expand our analysis beyond the Δ*S* = 0.1 gradient and investigate gradients of the same lateral size but with a varying spectral range and thus varying steepness. In total, we fabricated and analysed eight gradients with spectral ranges from 225 nm to 1,910 nm (Δ*S* = 0.05–0.4). Figure 2f displays the average maximum reflectance amplitude for the different gradients, with their spectral coverage visualized by the bar length. We find a considerable decrease in resonance amplitude with increasing spectral coverage, dropping from an average reflectance amplitude of 0.52 for the 225 nm coverage case to 0.15 for the 1,910 nm coverage case. To quantify this correlated behaviour, we introduce the scaling increment *ε*_*S*_ = Δ*S* × *P*/ΔL, calculated from the maximum scaling factor variation Δ*S*, unit cell periodicity *P* and spatial extent Δ*L* of the gradient metasurface. This metric offers an intuitive way to characterize the behaviour of the gradient, since it represents the change in scaling factor between two neighbouring unit cells. Extracted average reflectance amplitudes and *Q*-factors for different tilting angles *θ* (10°, 20° and 30°) are shown in Fig. 2g as a function of *ε*_*S*_. For a comparison, the performance of the monospectral metasurfaces for *θ* = 20° is shown as dashed lines. Although the average resonance amplitude and *Q*-factor decrease as *ε*_*S*_ increases, the spectral gradients can match the performance of the monospectral metasurfaces when *ε*_*S*_ ≤ 0.5 × 10^–3^, as highlighted for the case of *θ* = 20° (Fig. 2g). This insight into BICs is crucial for applications requiring both continuous spectral coverage and maximum resonance performance. It clearly demonstrates that the pseudoperiodicity does not have a negative impact if *ε*_*S*_ ≤ 0.5 × 10^–3^. Crucially, even in the parameter range where reflectance and *Q*-factor fall below the monospectral case (*ε*_*S*_ ≥ 0.5 × 10^–3^), spectral gradients can still provide considerable benefits, such as hyperspectral operation, while maintaining sufficient optical performance.

## Coupling gradients

Following the demonstration of spectral gradients, we now focus on spatially encoding the radiative losses of BIC-driven metasurfaces. As introduced in Fig. 1b, tuning of the ellipse opening angle *θ* can provide resonances with a wide range of *Q*-factors, following the characteristic inverse square relationship *Q* = 1/*α*^2^ = 1/sin^2^(*θ*) (Supplementary Fig. 1b)^45^. This precise resonance control allows for tailored interactions between the resonant mode and surrounding materials due to the proportionality of the *Q*-factor with the local electromagnetic field enhancement (FE)^49^ via FE^2^ ∝ *Q* (Supplementary Note 2).

**Fig. 3:**
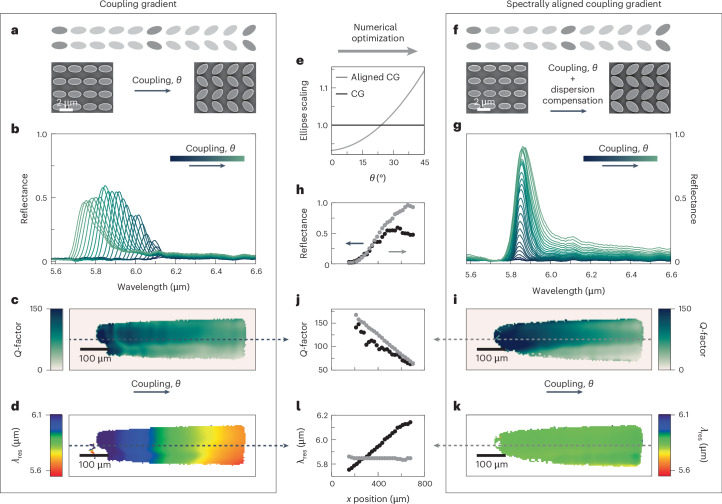
Coupling-gradient metasurface. **a**, Illustration of the coupling gradient oriented perpendicular to the excitation polarization, accompanied by SEM images of unit cells from the gradient’s start and end. **b**, Reflectance spectra from the gradients captured along the *x* axis. **c**, *Q*-factor map derived from temporal coupled mode theory for the coupling gradient. **d**, The associated resonance frequency map. **e**, Depiction of ellipse scaling ES dependent on *θ* for the coupling gradient (CG; black) and the spectrally aligned coupling gradient (grey). **f**, Illustration of the spectrally aligned gradient and SEM images of the gradient’s start and end. **g**, Reflectance spectra from the spectrally aligned coupling gradient, analogous to **b**. **h**, Reflectance amplitude comparison of the two gradients extracted from the temporal coupled mode theory fitted data. **i**, *Q*-factor map for the spectrally aligned coupling gradient. **j**, A comparison between both gradients taken along the dashed lines in **c** and **i**. **k**, Resonance wavelength map for the spectrally aligned gradient. **l**, A comparison of both gradients taken along the dashed lines in **d** and **k**.

Leveraging the concept of coupling gradients introduced above, we fabricated metasurfaces with continuously increasing values of *θ* ranging from 0° to 45° (Fig. 3a). The direction of the *θ* variation is chosen perpendicular to the excitation polarization, since this configuration provides better optical performance for spectral gradients (Supplementary Note 3 and Supplementary Fig. 11). The dimensions of the gradient are 650 × 150 µm^2^. Experimental reflectance spectra in Fig. 3b show overall increasing amplitudes for increasing *θ*, which we mainly attribute to the reduced susceptibility of lower-*Q* resonances to intrinsic material losses and fabrication defects (a more detailed analysis is in Supplementary Fig. 12). We attribute the decrease in amplitude for high *θ* to the quenching effect of the Rayleigh limit. Using temporal coupled mode theory (Supplementary Note 2 and Supplementary Fig. 13 for a schematic illustration), we fit the reflectance spectra of each pixel of our dataset to extract the characteristic resonance parameters (reflectance amplitude, resonance wavelength *λ*_res_ and *Q*-factor). The resulting *Q*-factor map in Fig. 3c (maximum reflectance map in Supplementary Fig. 14) aligns with our numerical design, showing a decrease in *Q*-factor with increasing *θ*. However, when changing the asymmetry, *λ*_res_ does not remain constant but undergoes a spectral shift of around 500 nm (Fig. 3d). This crosstalk between *Q* and *λ*_res_ is detrimental for applications, as it hinders the consistent spectral overlap with dispersive media like molecules and their vibration lines.

To overcome this challenge and facilitate the accurate implementation of dual-gradient metasurfaces, we introduce the concept of spectrally aligned coupling gradients using an additional ellipse scaling factor ES (Supplementary Fig. 2 for a sketch of the effect ES has on the geometric parameters). Instead of modifying all lateral unit cell parameters, ES alters solely the ellipses’ dimensions, *A* and *B*, while keeping *P*_*x*_ and *P*_*y*_ constant, allowing for local adjustments of the resonance wavelength without breaking the overall gradient metasurface pattern. Using extensive numerical simulations to determine ES values that offset the spectral shifts caused by *θ* (Fig. 3e and Supplementary Fig. 15), we then fabricated a spectrally aligned coupling gradient as shown in the sketch and SEM images at *θ* = 0° and 45° in Fig. 3f.

Experimental reflectance spectra for different asymmetries confirm the near-perfect spectral alignment of the resonances (Fig. 3g; maximum reflectance map in Supplementary Fig. 14). Additionally, the reflectance amplitude is substantially improved compared to the initial gradient, especially for large asymmetries (Fig. 3h). Likewise, the spectrally aligned coupling gradient delivers consistently higher values of the *Q*-factor for all tilting angles *θ* (Fig. 3i,j), and the resonance wavelength remains nearly constant at 5.85 µm, as shown in the resonance wavelength map in Fig. 3k and in the comparison plot in Fig. 3l. Supplementary Video 2 directly compares both coupling gradients frame by frame.

It is crucial to note that the relationship FE^2^ ∝ *Q* holds true only in lossless systems (Supplementary Note 2). In our silicon resonators, while material (intrinsic) losses are minimal, scattering losses are a substantial factor. These losses, arising from various sources such as surface roughness, variations in resonator size and imperfectly collimated excitation light, lead to discrepancies between our numerical and experimental findings. Specifically, this is evident in the deviation observed between the numerical results shown in Fig. 1d, where the reflectance amplitude is equal to 1 across all *Q*-factors, and the experimental results in Fig. 3g, where the reflectance amplitude is notably reduced, especially at higher *Q*-factors. Accounting for these losses, the peak field enhancement in our excited gradient is achieved at *θ* = 9° (Supplementary Fig. 16). Beyond this point, the coupling gradient demonstrates a continuous and smooth tuning of coupling strength and a linearly decreasing FE^2^ with the *Q*-factor (Supplementary Note 2). Future applications of our principle could potentially shift the point of highest field enhancement to smaller asymmetries, thereby aligning more closely with the ideal scenario. Supplementary Note 4 discusses the influence of scattering loss on the position of highest field enhancement in more detail. The ability to continuously scale the resonance line-width while maintaining its resonance frequency enables the full decoupling of spectral and coupling-strength tuning, providing the crucial prerequisite for realizing dual-gradient metasurfaces.

## Dual gradients for infrared absorption spectroscopy

**Fig. 4:**
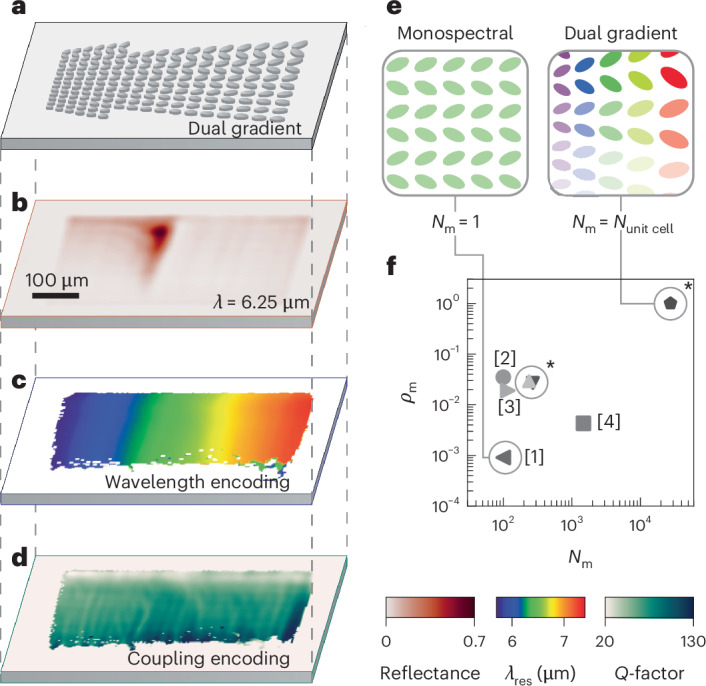
Dual-gradient metasurfaces and resonance density. **a**, Sketch of a dual-gradient metasurface with the spectral gradient along the *x* axis and the spectrally aligned coupling gradient along the *y* axis. **b**, Single-wavelength snapshot of the dual gradient with *S* = 0.95–1.25 and *θ* = 0–45° at 6.25 μm. **c**, Resonance wavelength map of the dual gradient showing continuous wavelength encoding along the *x* axis. **d**, *Q*-factor map of the dual gradient with decreasing values along the *y* axis. **e**, Illustrative comparison of distinct modes within conventional monospectral metasurfaces and a dual-gradient metasurface. In the monospectral metasurface, all unit cells are identical, resulting in a single supported resonance (*N*_m_ = 1). By contrast, each unit cell within the dual-gradient metasurface is unique, leading to *N*_m_ being equal to the total number of unit cells (*N*_unit cell_). **f**, A log–log plot of *N*_m_ against the resonance density (*ρ*_m_). Comparative works are marked with numbers: pixelated sensors [1] (ref. ^36^), radial BICs [2] (ref. ^58^), trapped rainbows [3] (ref. ^39^) and spectral-gradient metasurfaces [4] (ref. ^40^). Our work is highlighted with an asterisk (*), showcasing the spectral gradient and the coupling gradient at *ρ*_m_ = 2.9 × 10^–2^ and 2.7 × 10^–2^, respectively. The dual gradient is positioned at the top right, with *ρ*_m_ = 1.

A dual-gradient metasurface (Fig. 4a) is realized by building on the insights gained from the spectral and coupling gradients introduced above. Continuous spectral tuning is applied along the short axis (*P*_*x*_) of the unit cell, enabling the effective excitation of collective dipoles as discussed in Supplementary Fig. 11. Conversely, the tilting angle *θ* is scaled along the long axis of the unit cell (*P*_*y*_). We fabricated a dual-gradient measuring 600 × 450 µm^2^, with a spectral scaling factor *S* ranging from 0.95 to 1.25 (Δ*S* = 0.3) and *θ* from 0 to 45°. The optical performance of the dual gradient is illustrated by taking a single-wavelength reflectance image at 6.25 µm (Fig. 4b; additional wavelengths in Supplementary Fig. 17 and Supplementary Video 3), showcasing a narrow vertical strip indicating excellent spectral selectivity. The vertical strip increases in width for higher asymmetries, indicating precise control over the coupling strength.

Figure 4c shows the extracted resonance wavelength for each pixel, obtained through temporal coupled mode theory modelling, demonstrating a linear and continuous spectral coverage from 5.6 to 7.2 µm (Supplementary Fig. 18a,b). Notably, the resonance wavelength is unperturbed by different values of *θ*, as enabled by the spectral alignment procedure. Figure 4d shows the coupling-strength encoding, with *Q*-factors decreasing continuously from approximately 130 to 20 along the *y* axis as *θ* increases (Supplementary Fig. 18c,d for a cut along the *y* axis). By combining the wavelength and coupling-strength encodings highlighted in Fig. 4c,d, we demonstrate a dual-gradient metasurface that continuously maps both the wavelength and coupling parameter space in a unified nanophotonic system.

**Fig. 5:**
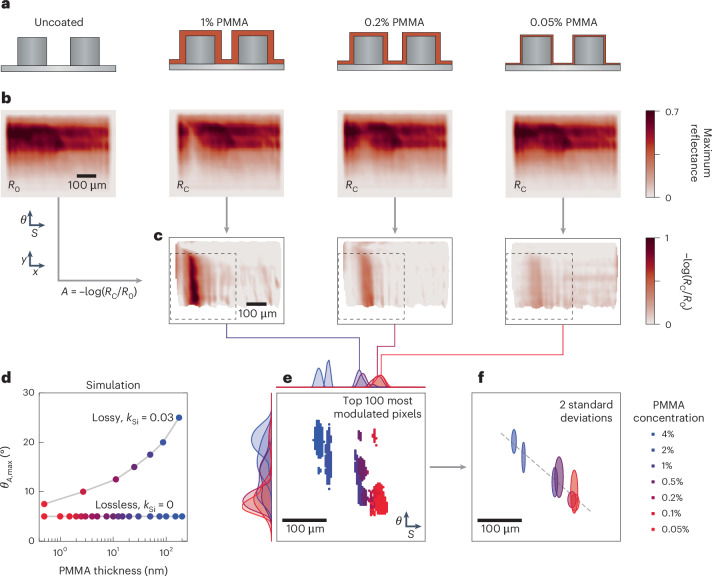
Dual-gradient metasurfaces for molecular sensing. **a**, Unit cell sketches of the dual gradient with varying thicknesses of an analyte coating (PMMA). **b**, Maximum reflectance maps of a dual gradient with *S* = 0.95–1.1 and *θ* = 0–45° for the different coating thicknesses following **a**. From left to right, the maps show the gradient with no PMMA coating, followed by layers created with 1%, 0.2% and 0.05% PMMA solutions. **c**, The absorbance signal for each pixel is calculated using –log(*R*_C_/*R*_0_). The absorbance due to the analyte’s vibrational fingerprint is evident within the left third of the dual gradient, with higher values corresponding to higher concentrations. **d**, Relationship between optimum sensing configuration and analyte concentration. The angle, *θ*_*A*,max_, where the relative absorbance *A* = –log(*R*_C_/*R*_0_) is maximal, is plotted against the analyte layer thickness. For an initially lossless system with *k*_Si_ = 0, *θ*_*A*,max_ remains at the lowest simulated angle of 5°. For *k*_Si_ = 0.03, which reflects the losses observed in our experiments (Supplementary Fig. 10), *θ*_*A*,max_ increases with the amount of analyte (more in Supplementary Note 7). **e**, A zoomed-in section of the dual gradient, outlined by the dashed black boxes in **c**, presents the 100 pixels with the highest absorbance for seven different coating thicknesses. The range starts with thick layers (4%) represented in blue and ends with thin layers (0.05%) in red. The kernel density estimation of the pixel distributions is plotted along both the *x* and *y* axes. **f**, The same zoomed-in section with the highest modulated pixels depicted as ellipses. The dimensions of the ellipses are set by twice the standard deviation in the *x* and *y* directions and are centred at the mean value. In grey is the linear regression line.

Delving deeper into the implications of dual gradients, we introduce the density of resonances *ρ*_res_ as a measure for the amount of information encoded within a metasurface. To ensure a wavelength-independent metric, we define *ρ*_m_ as the number of modes *N*_m_ divided by the number of unit cells *N*_unit cell_, that is, *ρ*_m_ = *N*_m_/*N*_unit cell_ (Supplementary Note 5 for more details). Conventional monospectral metasurfaces, as illustrated in Fig. 4e, typically feature only a single mode (*N*_m_ = 1). Consequently, *ρ*_m_ is low, for example *ρ*_m_ = 9.1 × 10^–4^ for a 100 × 100 µm^2^ metasurface^36^. While a shrinkage of the footprint will increase *ρ*_m_, a fundamental limit exists to the minimum pixel size that will sustain high-*Q* modes^38^. Conversely, in our spectral and coupling gradients, only one-dimensional chains of resonators share identical geometrical parameters, resulting in higher *ρ*_m_ values of 2.9 × 10^–2^ and 2.7 × 10^–2^, respectively. The dual gradients, as illustrated in Fig. 4e, show an even higher mode density, with each unit cell being unique within the gradient, and therefore encoding a distinct point in the 2D spectral–coupling parameter space. This pushes *ρ*_m_ to its theoretical maximum of 1 for metasurfaces with unit cells supporting a single mode. This leads to a total number of 22,800 modes across the dual gradient (27,500 modes in the dual gradient of Fig. 5). Such a high number of modes and *ρ*_m_ = 1 are unusual even for state-of-the-art metasurface designs. Even larger *ρ*_m_ values could be achieved by using unit cell geometries supporting more than one distinct mode. However, this would lead to spectral crosstalk between different spatial points within the gradient, substantially complicating data analysis and practical spectroscopic experiments. This mode density advancement is evident in the comparative analysis presented in Fig. 4f, where our dual-gradient design surpasses previous metasurface implementations, outperforming *ρ*_m_ in plasmonic gratings by a factor of 50, and exceeding dielectric metasurfaces by at least two orders of magnitude, as detailed in Supplementary Note 6 and Supplementary Table 1. In our methodology, *N*_m_ is considered the theoretical maximum number of supported resonances. However, practical limitations related to spatial and spectral resolution may reduce the actual number of distinguishable modes. Furthermore, our metric is generally valid only for extended metasurfaces, as isolated resonant nanostructures supporting at least one mode would always achieve *ρ*_m_ ≥ 1.

The dual gradient captures generalized information about light–matter coupling processes and consequently has potential applications in a wide variety of fields ranging from polaritonic coupling to quantum light emission and biochemical sensing. Here we focus on using dual gradients to obtain new functionalities for the surface-enhanced infrared absorption spectroscopy (SEIRAS) of molecular systems^15,50^. In addition to retrieving the conventional spectral fingerprint of the molecules, the integration of the coupling parameter into the gradient metasurface unlocks a new dimension of spectroscopy data, which is correlated with intrinsic analyte properties such as concentration. To reveal the relationship between analyte concentration and optimal sensing performance, we conducted a series of measurements on a dual-gradient metasurface (*S* = 0.95–1.1, *θ* = 0–45°; Supplementary Fig. 19; 27,500 distinct modes) with varying amounts of molecular analyte. We chose poly(methyl methacrylate) (PMMA) as the analyte because of its widespread use as a sensor benchmark^51–53^ and the straightforward control over the layer thickness through spin-coating of solutions with varying concentrations. Seven concentrations of PMMA dissolved in anisole ranging from 4% to 0.05% were prepared and spin-coated onto the metasurface at 3,000 rpm, resulting in layer thicknesses of approximately 200 nm (4%) to 1.5 nm (0.05%).

Figure 5a illustrates the varying thicknesses of the PMMA coating on top of the resonators; the uncoated structure is on the far left, followed by structures coated with decreasing concentrations of 1%, 0.2% and 0.05% PMMA solution. Maximum reflectance images of the dual gradient with the coatings from Fig. 5a are presented in Fig. 5b. The molecular fingerprint of PMMA can be clearly resolved by eye for the higher concentrations, where it appears as an area of reduced maximum reflectance within the left third of the dual gradient. Supplementary Fig. 20 further illustrates the molecular fingerprint within the *Q*-factor map for a 1% concentration. By calculating the relative absorbance of the analyte on a pixel-by-pixel basis via *A* = –log(*R*_C_/*R*_0_), where *R*_C_ and *R*_0_ represent the maximum reflectance of the coated and uncoated gradient metasurface, respectively, the molecular fingerprint of PMMA becomes evident for all concentrations (Fig. 5c; wavelength by wavelength comparison in Supplementary Videos 4 and 5). While we expect a correlation between overall absorbance modulation and analyte concentration, a closer look reveals an additional shift of the most sensitive pixels (highest *A*) along both the spectral and coupling-strength axes as the concentration varies. The spectral shift aligns well with the analyte-induced refractive index changes, but the coupling-strength shift needs further investigation.

Simulations across various analyte concentrations and ellipse tilt angles (Supplementary Note 7 and Supplementary Figs. 21 and 23) challenge the common belief that the highest *Q*-factors always yield the highest SEIRAS sensitivity. This behaviour becomes evident in Fig. 5d, which shows *θ*_*A*,max_ (that is, the ellipse tilt angle where the relative absorbance *A* is maximal) for varying analyte layer thicknesses from 0.5 to 200 nm. It reveals distinct trends of *θ*_*A*,max_ for both lossless (extinction coefficient *k*_Si_ = 0) and lossy (*k*_Si_ = 0.03) systems, where the value of *k*_Si_ is chosen to mirror the loss observed in our experimental data (Supplementary Fig. 10). For *k*_Si_ = 0, the smallest simulated angle and thus the highest *Q*-factor performs best regardless of analyte concentration, yielding a constant *θ*_*A*,max_. However, for *k*_Si_ = 0.03, *θ*_*A*,max_ increases substantially from 7.5° (high *Q*-factors) to 25° (low *Q*-factors) when the analyte layer thickness increases. These findings are confirmed by analytical modelling (Supplementary Note 7 and Supplementary Fig. 22).

Revisiting our experimental data, Fig. 5e shows the 100 most sensitive (highest absorbance modulation) pixels for each concentration within a subsection of the full gradient (marked area in Fig. 5c). As the analyte concentration decreases, these pixels shift towards larger scaling factors due to reduced spectral redshift. This refractive index sensitivity allows the dual gradient to provide a complementary source of information on the analyte besides the absorption-based sensing data. Moreover, a trend towards lower asymmetries (higher *Q*-factors) is visible when the concentration decreases, as evidenced by the mean values plotted for each concentration in Fig. 5f. These experimental results strongly support the numerical and analytical findings given above, demonstrating a clear shift towards lower *Q*-factors (and therefore lower optimal coupling strength) with increasing analyte concentration. Although determining and setting the ideal coupling strength is crucial for optimizing next-generation sensor designs, we believe this phenomenon has not yet been observed in metasurface sensors, since they do not typically resolve the coupling-strength dimension at all.

## Conclusions

In our study, we have investigated and established a framework for describing the working principles of spectral BIC gradients and discussed their limitations in terms of scaling increments of neighbouring unit cells. Our results on spectral gradients are of special importance in the context of miniaturizing optical systems with a possible key application of on-chip spectrometers, where the spectral gradient could be directly fabricated on top of, for example a complementary metal–oxide–semiconductor sensor, allowing the metasurface to act as a 2D equivalent of a grating or prism. This integration would result in a simplified fabrication process and substantial reduction in system size.

We have introduced the concept of coupling gradients as a method to map the *Q*-factor of a resonance (and consequently its field enhancement) both continuously and spatially. We believe this feature makes coupling gradients highly promising for studying emerging materials at the nanoscale, such as the density of photonic states in quantum systems within the framework of Fermi’s golden rule, and tuning the absorption of light in arbitrary target materials. For the latter, the equilibrium point between intrinsic losses within the material and radiative losses, known as critical coupling, is often the ideal state for applications as it allows for the highest field enhancement combined with maximum absorption. Our coupling-gradient method enables the continuous mapping of the entire coupling space, including the critical coupling point, without requiring prior detailed knowledge of the absorptive properties of the material under investigation.

Numerous studies using BIC metasurfaces have demonstrated enhancement of nonlinear properties and stimulated emission, as well as strong light–matter coupling in van der Waals materials or quantum dots^14,54,55^. Our dual-gradient metasurface can map these interactions continuously and within a compact area, which is crucial for research involving small footprint materials such as exfoliated 2D materials. Furthermore, recent trends in photocatalysis emphasize light-emitting diode (LED)-driven nanophotonic reactors as energy efficient alternatives to traditional thermocatalytic methods^6^. In such reactors, BICs have a high potential^56,57^ as they typically employ low-loss materials that ensure high absorbance within any catalytic material placed in close proximity. The introduction of dual gradients could streamline the optimization of these systems, offering a substantial advantage to discover and explore catalytic materials. In this context, we emphasize that the concept of dual gradients can be applied to the visible spectral range by simply adjusting the geometric parameters of the resonator (Supplementary Fig. 24). Finally, for molecular sensing, detection efficiency and miniaturization are important, but sometimes contrasting, aspects. Our dual gradient achieves a total number of distinct modes of 27,500, all within a compact area of 650 × 450 μm^2^. Our results show how the sensitivity of a sensor is linked to the *Q*-factor and the concentration of the analyte. But, while the common notion is that higher *Q*-factors lead to higher sensitivities, we show this is true only for lossless systems. In fact, we identify a considerable shift of the optimal *Q*-factor when the analyte concentration is changed. Our dual gradient, with its complete coverage of the coupling space, offers optimal sensitivity regardless of analyte concentration, degradation of the sensor over time or introduction of additional lossy materials like solvents.

## Methods

### Numerical methods

Our simulations were conducted using CST Studio Suite (Simulia), a commercial finite element solver. We configured the software for adaptive mesh refinement and periodic boundary conditions, operating in the frequency domain. The CaF_2_ substrate was modelled as loss-free and non-dispersive within our targeted wavelength range with a refractive index of 1.36. The refractive index of amorphous silicon was set to 3.32, based on near-infrared ellipsometric data.

### Sample fabrication

We deposited a 750 nm layer of amorphous silicon on a CaF_2_ substrates using plasma-enhanced chemical vapour deposition with the PlasmaPro 100 system (Oxford Instruments). The nanostructuring process started with spin-coating a 400 nm layer of positive electron-beam resist, ZEP520A (Zeon Corporation), followed by a conductive polymer coating using ESPACER (Showa Denko K.K.). Electron-beam lithography was performed using an eLINE Plus system (Raith) at 20 kV with a 20 µm aperture. The patterned films were developed in an amyl acetate bath, followed by a bath of methyl isobutyl ketone and isopropyl alcohol (1:9 ratio). A 60 nm chromium layer was then deposited, and the resist was lifted off using Microposit Remover 1165 (Microresist). The remaining chromium served as an etching mask for the subsequent reactive ion etching process, which used SF_6_ and argon gases. Finally, the chromium mask was removed using TechniEtch Cr01 (MicroChemicals).

### Optical characterization

Optical measurements were performed with a Spero spectral imaging mid-infrared microscope (Daylight Solutions), as illustrated in Supplementary Fig. 5a. The microscope featured a ×4 magnification objective (numerical aperture = 0.15) and provided a 2 mm^2^ field of view of 480 pixel × 480 pixel, and a pixel size of approximately 4 × 4 µm^2^. For an in-depth exploration on why the angles of incoming light have neglectable effects on the resonances, Supplementary Note 8 and Supplementary Figs. 25–27 contain further details. This system was equipped with three tunable quantum cascade lasers covering a wavelength range of 5.6–10.5 μm and offering a spectral resolution of 2 cm^−1^. The lasers emitted linearly polarized light, essential for our measurements.

### Molecular sensing

We coated the dual gradient with different concentrations of PMMA (495 K, diluted in anisole), ranging from 0.05% to 4%. Each solution was uniformly applied to the metasurface through spin-coating at 3,000 rpm for 1 min. Following the coating, the metasurface was baked at 180 °C for 3 min to ensure the PMMA layer was fully solidified. To remove the PMMA layer between each measurement run, the PMMA films were sequentially dissolved using an acetone bath, followed by an isopropyl alcohol bath. As an additional cleaning step, the sample was UV cleaned for 30 min, followed by another round of acetone and isopropyl alcohol baths to remove any residual substances.

The thickness of the PMMA layers was estimated based on ellipsometric data obtained for 0.1%, 0.25% and 4% solutions. We fitted a polynomial curve to this data with the following parameters: *d* = 1.237*c*^3^ + 4.773*c*^2^ + 10.88*c* + 0.9635, with *d* as the thickness in nanometres and *c* as the concentration as a percent. This leads to layer thicknesses ranging from 1.5 nm (0.05%) to 200 nm (4%). The measurements were conducted using an HS-190 J.A. Woollam VASE ellipsometer.

## Online content

Any methods, additional references, Nature Portfolio reporting summaries, source data, extended data, supplementary information, acknowledgements, peer review information; details of author contributions and competing interests; and statements of data and code availability are available at 10.1038/s41565-024-01767-2.

## Supplementary information

Supplementary Information Supplementary Notes 1–8, Table 1 and Figs. 1–27.

Supplementary Video 1 Reflectance video of the three monospectral metasurfaces with scaling factors of 1.01, 1.05 and 1.09, and the spectral gradient of *S* = 1.0–1.1, shown in Fig. 2. The reflectance colour code spans values from 0 to 0.7, the start frame is at 5,672 nm and the end frame is at 6,569 nm.

Supplementary Video 2 Reflectance video of the coupling gradient (top) and the spectrally aligned coupling gradient (bottom), discussed in Fig. 3. The reflectance colour code spans values from 0 to 0.9, the start frame is at 5,608 nm and the end frame is at 6,319 nm.

Supplementary Video 3 Reflectance video of the dual gradient discussed in Fig. 4. The reflectance colour code spans values from 0 to 0.7, the start frame is at 5,574 nm and the end frame is at 7,153 nm.

Supplementary Video 4 Reflectance video of the uncoated dual gradient discussed in Fig. 5. The reflectance colour code spans values from 0 to 0.7, the start frame is at 5,574 nm and the end frame is at 6,588 nm.

Supplementary Video 5 Reflectance video of the dual gradient discussed in Fig. 5, coated with a 1% solution of PMMA. The reflectance colour code spans values from 0 to 0.7, the start frame is at 5,574 nm and the end frame is at 6,588 nm.

## Data Availability

The data that support the findings of this study are available via *Zenodo* at 10.5281/zenodo.13254205 (ref. ^59^).

## References

[1] Meher, N. & Sivakumar, S. A review on quantum information processing in cavities. *Eur. Phys. J. Plus* **137**, 985 (2022).

[2] Oldenburg, S. J., Averitt, R. D., Westcott, S. L. & Halas, N. J. Nanoengineering of optical resonances. *Chem. Phys. Lett.* **288**, 243–247 (1998).

[3] Kivshar, Y. & Miroshnichenko, A. Meta-optics with Mie resonances. *Opt. Photon. News* **28**, 24–31 (2017).

[4] Atwater, H. A. & Polman, A. Plasmonics for improved photovoltaic devices. *Nat. Mater.* **9**, 205–213 (2010). 20168344 10.1038/nmat2629

[5] Ricci, F. et al. A chemical nanoreactor based on a levitated nanoparticle in vacuum. *ACS Nano* **16**, 8677–8683 (2022). 35580358 10.1021/acsnano.2c01693

[6] Yuan, Y. et al. Earth-abundant photocatalyst for H_2_ generation from NH_3_ with light-emitting diode illumination. *Science* **378**, 889–893 (2022). 36423268 10.1126/science.abn5636

[7] Dousse, A. et al. Ultrabright source of entangled photon pairs. *Nature* **466**, 217–220 (2010). 20613838 10.1038/nature09148

[8] Orieux, A., Versteegh, M. A. M., Jöns, K. D. & Ducci, S. Semiconductor devices for entangled photon pair generation: a review. *Rep. Prog. Phys.* **80**, 076001 (2017). 28346219 10.1088/1361-6633/aa6955

[9] García-Guirado, J., Svedendahl, M., Puigdollers, J. & Quidant, R. Enhanced chiral sensing with dielectric nanoresonators. *Nano Lett.* **20**, 585–591 (2020). 31851826 10.1021/acs.nanolett.9b04334

[10] Hu, H. et al. Nanoscale Bouligand multilayers: giant circular dichroism of helical assemblies of plasmonic 1D nano-objects. *ACS Nano* **15**, 13653–13661 (2021). 34375085 10.1021/acsnano.1c04804

[11] Duan, J. et al. Twisted nano-optics: manipulating light at the nanoscale with twisted phonon polaritonic slabs. *Nano Lett.* **20**, 5323–5329 (2020). 32530634 10.1021/acs.nanolett.0c01673

[12] Zhang, Q. et al. Interface nano-optics with van der Waals polaritons. *Nature* **597**, 187–195 (2021). 34497390 10.1038/s41586-021-03581-5

[13] Dang, N. H. M. et al. Tailoring dispersion of room-temperature exciton-polaritons with perovskite-based subwavelength metasurfaces. *Nano Lett.* **20**, 2113–2119 (2020). 32074449 10.1021/acs.nanolett.0c00125

[14] Weber, T. et al. Intrinsic strong light–matter coupling with self-hybridized bound states in the continuum in van der Waals metasurfaces. *Nat. Mater.* **22**, 970–976 (2023). 37349392 10.1038/s41563-023-01580-7 PMC10390334

[15] Neubrech, F., Huck, C., Weber, K., Pucci, A. & Giessen, H. Surface-enhanced infrared spectroscopy using resonant nanoantennas. *Chem. Rev.* **117**, 5110–5145 (2017). 28358482 10.1021/acs.chemrev.6b00743

[16] Etezadi, D. et al. Nanoplasmonic mid-infrared biosensor for in vitro protein secondary structure detection. *Light. Sci. Appl.* **6**, e17029 (2017). 30167280 10.1038/lsa.2017.29 PMC6062318

[17] Hentschel, M., Schäferling, M., Weiss, T., Liu, N. & Giessen, H. Three-dimensional chiral plasmonic oligomers. *Nano Lett.* **12**, 2542–2547 (2012). 22458608 10.1021/nl300769x

[18] Hentschel, M., Dregely, D., Vogelgesang, R., Giessen, H. & Liu, N. Plasmonic oligomers: the role of individual particles in collective behavior. *ACS Nano* **5**, 2042–2050 (2011). 21344858 10.1021/nn103172t

[19] Liu, S. D. et al. Metasurfaces composed of plasmonic molecules: hybridization between parallel and orthogonal surface lattice resonances. *Adv. Opt. Mater*. **8**, 1901109 (2020).

[20] Reshef, O. et al. Multiresonant high-*Q* plasmonic metasurfaces. *Nano Lett.* **19**, 6429–6434 (2019). 31454252 10.1021/acs.nanolett.9b02638

[21] Chen, K., Adato, R. & Altug, H. Dual-band perfect absorber for multispectral plasmon-enhanced infrared spectroscopy. *ACS Nano* **6**, 7998–8006 (2012). 22920565 10.1021/nn3026468

[22] Gottheim, S., Zhang, H., Govorov, A. O. & Halas, N. J. Fractal nanoparticle plasmonics: the Cayley tree. *ACS Nano* **9**, 3284–3292 (2015). 25727720 10.1021/acsnano.5b00412

[23] Wallace, G. Q. & Lagugné-Labarthet, F. Advancements in fractal plasmonics: structures, optical properties, and applications. *Analyst* **144**, 13–30 (2019). 10.1039/c8an01667d 30403204

[24] Zhou, Y., Guo, S., Overvig, A. C. & Alù, A. Multiresonant nonlocal metasurfaces. *Nano Lett.* **23**, 6768–6775 (2023). 37307588 10.1021/acs.nanolett.3c00772

[25] Chen, H. T., Taylor, A. J. & Yu, N. A review of metasurfaces: physics and applications. *Rep. Prog. Phys.* **79**, 076401 (2016). 27308726 10.1088/0034-4885/79/7/076401

[26] Cortés, E. et al. Optical metasurfaces for energy conversion. *Chem. Rev.* **122**, 15082–15176 (2022). 35728004 10.1021/acs.chemrev.2c00078 PMC9562288

[27] Karst, J. et al. Electrically switchable metallic polymer nanoantennas. *Science* **374**, 612–616 (2021). 10.1126/science.abj3433 34709910

[28] Ding, F., Pors, A. & Bozhevolnyi, S. I. Gradient metasurfaces: a review of fundamentals and applications. *Rep. Prog. Phys.* **81**, 026401 (2018). 28825412 10.1088/1361-6633/aa8732

[29] Chen, W. T., Zhu, A. Y. & Capasso, F. Flat optics with dispersion-engineered metasurfaces. *Nat. Rev. Mater.* **5**, 604–620 (2020).

[30] Gan, Q. et al. Experimental verification of the rainbow trapping effect in adiabatic plasmonic gratings. *Proc. Natl Acad. Sci. USA* **108**, 5169–5173 (2011). 21402936 10.1073/pnas.1014963108 PMC3069179

[31] Xu, Z., Shi, J., Davis, R. J., Yin, X. & Sievenpiper, D. F. Rainbow trapping with long oscillation lifetimes in gradient magnetoinductive metasurfaces. *Phys. Rev. Appl.* **12**, 024043 (2019).

[32] Azzam, S. I. & Kildishev, A. V. Photonic bound states in the continuum: from basics to applications. *Adv. Opt. Mater.* **9**, 16–24 (2021).

[33] Watanabe, K., Devi, H. R., Iwanaga, M. & Nagao, T. Vibrational coupling to quasi‐bound states in the continuum under tailored coupling conditions. *Adv. Opt. Mater.* **12**, 2301912 (2024).

[34] Koshelev, K., Favraud, G., Bogdanov, A., Kivshar, Y. & Fratalocchi, A. Nonradiating photonics with resonant dielectric nanostructures. *Nanophotonics* **8**, 725–745 (2019).

[35] Kodigala, A. et al. Lasing action from photonic bound states in continuum. *Nature* **541**, 196–199 (2017). 28079064 10.1038/nature20799

[36] Tittl, A. et al. Imaging-based molecular barcoding with pixelated dielectric metasurfaces. *Science* **360**, 1105–1109 (2018). 29880685 10.1126/science.aas9768

[37] Jahani, Y. et al. Imaging-based spectrometer-less optofluidic biosensors based on dielectric metasurfaces for detecting extracellular vesicles. *Nat. Commun.* **12**, 3246 (2021). 34059690 10.1038/s41467-021-23257-y PMC8167130

[38] Liu, Z. et al. High-*Q* quasibound states in the continuum for nonlinear metasurfaces. *Phys. Rev. Lett.* **123**, 253901 (2019). 31922806 10.1103/PhysRevLett.123.253901

[39] Zhou, L. et al. Super-resolution displacement spectroscopic sensing over a surface “rainbow”. *Engineering* **17**, 75–81 (2022). 38149108 10.1016/j.eng.2022.03.018 PMC10751035

[40] Jangid, P. et al. Spectral tuning of high‐harmonic generation with resonance‐gradient metasurfaces. *Adv. Mater.* **36**, 2307494 (2023). 10.1002/adma.202307494 37933748

[41] Wang, S. et al. Broadband achromatic optical metasurface devices. *Nat. Commun.* **8**, 187 (2017). 28775300 10.1038/s41467-017-00166-7 PMC5543157

[42] Chen, W. T. et al. A broadband achromatic metalens for focusing and imaging in the visible. *Nat. Nanotechnol.* **13**, 220–226 (2018). 29292382 10.1038/s41565-017-0034-6

[43] Overvig, A. C., Malek, S. C. & Yu, N. Multifunctional nonlocal metasurfaces. *Phys. Rev. Lett.* **125**, 017402 (2020). 32678662 10.1103/PhysRevLett.125.017402

[44] Kühne, J. et al. Fabrication robustness in BIC metasurfaces. *Nanophotonics* **10**, 4305–4312 (2021).

[45] Koshelev, K., Lepeshov, S., Liu, M., Bogdanov, A. & Kivshar, Y. Asymmetric metasurfaces with high-*Q* resonances governed by bound states in the continuum. *Phys. Rev. Lett.* **121**, 193903 (2018). 30468599 10.1103/PhysRevLett.121.193903

[46] Cai, W. & Shalaev, V. *Optical Metamaterials: Fundamentals and Applications* (Springer, 2010).

[47] Barkey, M. et al. Pixelated high-Q metasurfaces for in situ biospectroscopy and artificial intelligence-enabled classification of lipid membrane photoswitching dynamics. *ACS Nano* **18**, 11644–11654 (2024). 38653474 10.1021/acsnano.3c09798 PMC11080459

[48] Gölz, T. et al. Revealing mode formation in quasi-bound states in the continuum metasurfaces via near-field optical microscopy. *Adv. Mater.*10.1002/adma.202405978 (2024). 10.1002/adma.202405978 39092689

[49] Yoon, J. W., Song, S. H. & Magnusson, R. Critical field enhancement of asymptotic optical bound states in the continuum. *Sci. Rep.* **5**, 18301 (2015). 26673548 10.1038/srep18301 PMC4682140

[50] Altug, H., Oh, S. H., Maier, S. A. & Homola, J. Advances and applications of nanophotonic biosensors. *Nat. Nanotechnol.* **17**, 5–16 (2022). 35046571 10.1038/s41565-021-01045-5

[51] Adato, R., Artar, A., Erramilli, S. & Altug, H. Engineered absorption enhancement and induced transparency in coupled molecular and plasmonic resonator systems. *Nano Lett.* **13**, 2584–2591 (2013). 23647070 10.1021/nl400689q

[52] Paggi, L. et al. Over-coupled resonator for broadband surface enhanced infrared absorption (SEIRA). *Nat. Commun.* **14**, 4814 (2023). 37558692 10.1038/s41467-023-40511-7 PMC10412556

[53] Aigner, A. et al. Plasmonic bound states in the continuum to tailor light-matter coupling. *Sci. Adv.* **8**, add4816 (2022). 10.1126/sciadv.add4816 PMC9733921 36490330

[54] Kravtsov, V. et al. Nonlinear polaritons in a monolayer semiconductor coupled to optical bound states in the continuum. *Light Sci. Appl*. **9**, 56 (2020). 10.1038/s41377-020-0286-z PMC7145813 32284858

[55] Wang, X. et al. Controlling light absorption of graphene at critical coupling through magnetic dipole quasi-bound states in the continuum resonance. *Phys. Rev. B* **102**, 155432 (2020).

[56] Yuan, L. et al. A quasi-bound states in the continuum dielectric metasurface-based antenna-reactor photocatalyst. *Nano Lett.* **24**, 172–179 (2024). 38156648 10.1021/acs.nanolett.3c03585

[57] Hu, H. et al. Catalytic metasurfaces empowered by bound states in the continuum. *ACS Nano* **16**, 13057–13068 (2022). 35953078 10.1021/acsnano.2c05680 PMC9413421

[58] Kühner, L. et al. Radial bound states in the continuum for polarization-invariant nanophotonics. *Nat. Commun.* **13**, 4992 (2022). 36008419 10.1038/s41467-022-32697-z PMC9411165

[59] Aigner, A., Weber, T., Wester, A., Maier, S. A. & Tittl, A. Data supporting publication: Continuous spectral and coupling-strength encoding with dual-gradient metasurfaces. *Zenodo*10.5281/zenodo.13254205 (2024). 10.1038/s41565-024-01767-2 PMC11638065 39187580

