# Supplementary material for: Continuous spectral and coupling-strength encoding with dual-gradient metasurfaces
Source: Nat Nanotechnol. 2024 Aug 26;19(12):1804–12. doi: 10.1038/s41565-024-01767-2 (PMC11638065; doi:10.1038/s41565-024-01767-2)
Supplement: Supplementary file 1 — Supplementary Notes 1–8, Table 1 and Figs. 1–27. [file 41565_2024_1767_MOESM1_ESM.pdf]

nature nanotechnology

Supplementary information https://doi.org/10.1038/s41565-024-01767-2

Continuous spectral and coupling-strength encoding with dual-gradient metasurfaces

In the format provided by the authors and unedited

Table of contents

Supplementary Notes 2
Note 1: Comparison between experimental spectra and simulations 2
Note 2: Temporal coupled mode theory and fitting 2
Note 3: Effect of the unit cell arrangement in a scaling gradient 4
Note 4: Influence of scattering loss on the position of highest field enhancement 5
Note 5: Calculating the resonance density 6
Note 6: Mode density comparison to literature 7
Note 7: Numerical and analytical study on concentration dependent sensitivity 8
Note 8: Influence of the incident angle 10
Supplementary Tables 12
Table 1: Comparison of the total number of resonances and the resonance density 12
Supplementary Videos 13
Supplementary Video 1: Spectral gradient 13
Supplementary Video 2: Coupling gradient 13
Supplementary Video 3: Dual gradient 13
Supplementary Video 4: Dual gradient for molecular sensing, uncoated 13
Supplementary Video 5: Dual gradient for molecular sensing, coated 13
Supplementary Figures 14
Supplementary References 32

Supplementary Notes

Note 1: Comparison between experimental spectra and simulations

For a direct comparison, it is crucial to first address the impact of losses on our resonant system. In general, the total losses. In general, the total losses γ_{tot} can be written as the sum of individual contributions:

$$\gamma_{\text{tot}} = \gamma_{\text{rad}} + \gamma_{\text{int}} + \gamma_{\text{scat}} = \gamma_{\text{rad}} + \gamma_{\text{para}} \quad (1)$$

where $\gamma_{\text{rad}}, \gamma_{\text{int}},$ and γ_{scat} denote the radiative, intrinsic, and scattering losses, respectively. $\gamma_{\text{para}} = \gamma_{\text{int}} + \gamma_{\text{scat}}$ represents the combined parasitic losses. As highlighted in Supplementary Note 2, parasitic losses are inevitable in real-world applications. Importantly, when modeling these losses (as discussed in Supplementary Note 2) distinguishing between intrinsic or scattering losses is, in fact, not necessary, as both can be collectively represented by γ_{para} in the TCMT model (Further discussed in Minor Point #10). In our simulations, we incorporate the influence of γ_{para} on the BIC metasurface resonance by introducing an imaginary part of the refractive index of Si.

In **Figure S10a**, we simulate the reflectance for $\theta = 20°$ and $S = 1.01$ for varying values of the imaginary part of the refractive index of Si (k_{Si}) from 0 to 0.03. Although the resonance position remains unchanged, its amplitude decreases. When comparing the resonance with $k_{\text{Si}} = 0.03$ to the experimentally obtained results (spectral gradient of **Figure 2a-e**, $\theta = 20°$, $S = 1.01$) in **Figure S10b**, we observe a very good agreement between the experimental and numerical results, both in resonance shape and spectral position. The discrepancy in resonance position is only 16 nm, which corresponds to a spectral variation of only 0.27% for the mid-infrared operating wavelength of 5.9 µm. Incorporating the loss estimation enables a more accurate comparison between experimental and simulation outcomes.

Note 2: Temporal coupled mode theory and fitting

Temporal coupled mode theory (TCMT) is a straightforward and well-established formalism for describing coupled resonances in cavities that are also connected to the far field[1,2]. In our study, we employ a model consisting of a single resonator coupled to two ports, enabling transmission and reflection (**Figure S13**). The excitation occurs through port 1, represented as $\boldsymbol{s}_{+} = (s_{1+}, 0)^{\text{T}}$ and the output can be described as $\boldsymbol{s}_{-} = (s_{1-}, s_{2-})^{\text{T}}$, where s_{1-} denotes the reflected and s_{2-} the transmitted waves. The time evolution of the resonant mode amplitude $a(t)$ of our mirror-symmetric metasurface is described by the following coupled equations:

$$\frac{\mathrm{d}a(t)}{\mathrm{d}t} = (i\omega_0 - \gamma_{\text{tot}})\, a(t) + \boldsymbol{\kappa}^{\text{T}} \boldsymbol{s}_{+} \quad (2)$$

$$\boldsymbol{s}_{-} = C\boldsymbol{s}_{+} + a(t)\boldsymbol{\kappa} \quad (3)$$

Here, the resonance frequency of the mode is denoted by ω_0, the total damping rate consists of the radiative damping rate and parasitic damping rates such as material absorption and scattering losses $\gamma_{\text{tot}} = \gamma_{\text{rad}} + \gamma_{\text{int}} + \gamma_{\text{scat}} = \gamma_{\text{rad}} + \gamma_{\text{para}}$. The coupling between the ports and the mode is governed by the radiative damping rate $\boldsymbol{\kappa} = \left(\sqrt{\gamma_{\text{rad}}}, \sqrt{\gamma_{\text{rad}}}\right)^{\text{T}}$, whereas the non-resonant coupling between the port is described by the unitary matrix C

$$C = e^{i\varphi} \cdot \begin{pmatrix} r_0 & it_0 \\ it_0 & r_0 \end{pmatrix} \quad (4)$$

where r_0 (t_0) describes the background reflection (transmission) with $r_0^2 + t_0^2 = 1$ and φ is a global phase, together giving rise to asymmetric Fano lineshapes.

Assuming a time-harmonic mode amplitude ($a(t) \sim e^{i\omega t} \rightarrow \dot{a} = i\omega a$), we can rewrite equations (2) and (3) and solve for the reflection coefficient

$$r(\omega) = \frac{s_{1-}}{s_{1+}} = e^{i\varphi} r_0 + \frac{\gamma_{\mathrm{rad}}}{i(\omega - \omega_0) + \gamma_{\mathrm{tot}}} \quad (5)$$

The reflectance signals can thus be fitted by

$$R(\omega) = |r(\omega)|^2 \quad (6)$$

and the total Q-factor can be calculated from $Q_{\mathrm{tot}} = \frac{\omega_0}{2\gamma_{\mathrm{tot}}}$.

Next, we calculate the mode amplitude a as

$$a(\omega) = \frac{\sqrt{\gamma_{\mathrm{rad}}} s_{1+}}{i(\omega - \omega_0) + \gamma_{\mathrm{para}} + \gamma_{\mathrm{rad}}} \quad (7)$$

Because a is normalized such that $|a|^2$ denotes the total energy in the cavity, the electric field enhancement can be expressed as

$$\left|\frac{E}{E_0}\right|^2 \sim \left|\frac{a_{\mathrm{max}}}{s_{1+}}\right|^2 = \frac{\gamma_{\mathrm{rad}}^2}{\left(\gamma_{\mathrm{para}} + \gamma_{\mathrm{rad}}\right)^2} \quad (8)$$

Where $a_{\mathrm{max}} = a(\omega = \omega_0)$ is the maximum amplitude at resonance frequency.

This is the general expression for a metasurface exhibiting both radiative and parasitic losses.

For lossless systems or at the critical coupling condition ($\gamma_{\mathrm{para}} = \gamma_{\mathrm{rad}}$), the dependence of the field enhancement simplifies to

$$\left|\frac{E}{E_0}\right|^2 \sim \frac{1}{\gamma} \sim Q \quad (9)$$

Where Q is either the radiative Q-factor (lossless case) or total Q-factor at the critical coupling condition. Thus, the Q-factor of our metasurface has direct and linear influence on the electric near-field enhancement.

Note 3: Effect of the unit cell arrangement in a scaling gradient

At its core, the spectral gradient can be interpreted as a concatenation of individual resonant chains, all with slightly offset resonance wavelengths. A single chain of resonators has been shown to produce a weak but observable BIC resonance when the resonators are positioned such that their effective dipole moments align, allowing them to form a collective dipole[3,4]. Therefore, analyzing and understanding the impact on the unit cells' orientation in these chains within the gradient is crucial for the optical performance.

To investigate this effect in our spectral-gradient metasurfaces, we fabricated them in two configurations: one with scaling perpendicular to the excitation polarization (**Figure S11a**), as shown in **Figure 2**, and another with scaling parallel to the excitation polarization (**Figure S11b**). In our tilted double-ellipse design, the resonance emerges due to a net dipole moment along the unit cells' long axis indicated by the green arrow in **Figure S11a**. This is generated by two opposing individual dipoles (black arrows) within the ellipses when the tilting angle θ is non-zero. Consequently, a collective excitation occurs when the scaling is perpendicular to the excitation polarization. However, when the scaling is parallel to the excitation polarization, as depicted in **Figure S11b**, a collective dipole cannot form easily. This is due to the y-alignment offset of unit cells and the spectral shift of the dipolar moments along the coupling direction, which results in poor spectral overlap.

We experimentally tested both configurations and further examined the influence of gradient steepness, essentially the total spectral range within a spectral gradient of a given dimension. For this, we fabricated spectral gradients measuring 600x150 μm^2 with varied spectral ranges, spanning 225 nm to 1910 nm. **Figure S11c** (equivalent to **Figure 2g**) and **Figure S11d** display the average maximal reflectance amplitude for the gradients of different steepness in the two configurations. Strikingly, gradients with scaling normal to the excitation polarization exhibit a significantly better performance, for some spectral ranges more than twice the amplitude. This confirms the importance of the proper alignment of unit cells, ensuring effective dipole moment coupling, for optimal gradient performance.

Furthermore, the ε_S-dependency of amplitude and Q-factor for both configurations is plotted in **Figure S11e,f**. Solid lines represent gradients with scaling normal to the excitation polarization revealing their superior performance, while the dashed red lines show the results for metasurfaces with scaling parallel to the excitation polarization.

Note 4: Influence of scattering loss on the position of highest field enhancement

As discussed in **Supplementary Note 2**, in lossless systems, or at the critical coupling position the field enhancement is proportional to the Q-factor via $FE^2 \sim Q$. This implies the highest field enhancement coincides with the maximal Q-factor. However, this relationship becomes more complex when considering intrinsic and scattering losses, as further detailed in **Supplementary Note 2**.

In scenarios involving losses, the point of maximal field enhancement shifts to a lower Q-factor, known as the critical coupling point where $\gamma_{\text{int}} = \gamma_{\text{rad}}$. At this point, both resonance amplitude and absorption are maximized. While this phenomenon is extensively studied in lossy systems like plasmonic metasurfaces, it is less frequently addressed in dielectric systems, where intrinsic losses are typically minimal. Nevertheless, dielectric metasurfaces encounter additional non-intrinsic loss channels, collectively termed as scattering losses γ_{scat} in **Supplementary Note 2**. These losses arise from various sources such as resonator size variations, surface roughness, material inhomogeneities, finite array size, limited illumination area, and imperfect light collimation.

The point of highest resonance amplitude and thus field enhancement within our gradient is determined by specific fabrication qualities and the measurement setup. To accurately identify this point, we analyzed spectra along the gradient, dividing the gradient into 31 subsections, each representing a θ range of approximately 1.45° of the total 45°. The outermost sections were excluded due to scattering losses caused by the proximity to the edges. Given that structural quality, surface roughness, intrinsic losses, and losses from non-collimated excitations remain consistent across the gradient, we treated the parasitic losses, the combined intrinsic and scattering losses $\gamma_{\text{para}} = \gamma_{\text{int}} + \gamma_{\text{scat}}$, as constant. **Figure S16a** displays all spectra with their fits, demonstrating a good agreement across all θ values. Note that this fitting procedure with a shared parameter γ_{para} is computationally way more demanding than single spectra fits, as it is based on iteratively fitting all spectra in order to minimize the error for γ_{para}. **Figure S16b** illustrates the θ-dependent parasitic and radiative losses, and **Figure S16c** presents the corresponding Q-factors. The critical point of our analysis is the crossing of radiative and parasitic losses as well as radiative and parasitic Q-factors, indicating a critically coupled system with maximal field enhancement and highest coupling to adjacent materials. In our spectrally-aligned coupling gradient, this critical point is approximately at $\theta = 9°$.

This 9° deviation from the ideal scenario, where the highest field enhancement is near 0°, we attribute to our fabrication techniques and measurement setup. While intrinsic losses of silicon in the mid-infrared range are negligible, we assume that the primary source of parasitic losses stems from our measurement setup, particularly the non-perfectly collimated light.

It is important to clarify that these limitations are not fundamental and can be overcome in future applications of coupling-gradient metasurfaces. The exact position of this critical point may vary depending on intrinsic losses of the resonator material or any material of interest in proximity. For our current results, this implies that the effective coupling gradient begins not at the very end of the gradient ($\theta = 0°$) but slightly inward, around 9°. Beyond this point, for the remaining 36° θ-sweep, we observe a continuous, smooth coupling sweep, as visible in **Figure S16b**.

Finally, it is crucial to recognize that the point of highest field enhancement is not universally the most desirable. In applications like molecular sensing, high sensitivity does not necessarily correlate with the point of strongest field enhancement. While a robust field enhancement suggests strong coupling between the analyte and the resonator, a resonance that is sensitive to analyte-induced losses is often more critical. Hence, high sensitivity sensors tend to favor higher Q-factors over maximum field enhancement.

Note 5: Calculating the resonance density

In the spectral gradient, both P_x and P_y are scaled by the same factor S along the x-direction. The number of columns along the y-direction will vary depending on the scaling factor at each point along the x-axis. The number of unit cells $N_{\text{unit cell y}}$ within a column of the metasurface with height L_y for a specific scaling factor S can be calculated via

$$N_{\text{unit cell y}}(S) = \left\lfloor \frac{L_y}{S \cdot P_y} \right\rfloor (10)$$

with "⌊ ⌋" denoting the floor function, which rounds off to the nearest integer, ensuring that only whole unit cells within the border of the metasurface are counted.

Since the scaling changes linearly along the x-axis, one can calculate the number of unit cells along the x-axis by considering the average scaling factor. Let S_{start} and S_{end} be the start and end values of the scaling factor, respectively. The average scaling factor S_{avg} is given by $S_{\text{avg}} = \frac{1}{2} \cdot (S_{\text{start}} + S_{\text{end}})$.

Then, the number of unit cells along the x-axis is calculated by dividing the total length of the metasurface in x-direction (L_x) by the pitch in the x-direction scaled by the average scaling factor resulting in

$$N_{\text{unit cell x}} = \left\lfloor \frac{L_x}{S_{\text{avg}} \cdot P_x} \right\rfloor (11).$$

The total number of unit cells is obtained by integrating $N_{\text{unit cell y}}(S)$ over the range of scaling factors:

$$N_{\text{unit cell}} = \int_{S_{\text{start}}}^{S_{\text{end}}} N_{\text{unit cell y}}(S)\, \mathrm{d}S \ (12)$$

Given the linear variation of S, this integral simplifies to

$$N_{\text{unit cell}} = N_{\text{unit cell}} \cdot N_{\text{unit cell y,avg}}.$$

With $N_{\text{y,avg}}$ as the estimated average number of unit cells along the y-axis given by $N_{\text{y,avg}} = \frac{1}{2} \cdot \left(N_{\text{unit cell y,start}} + N_{\text{unit cell y,end}}\right) = \left\lfloor \frac{1}{2} \cdot \left(\frac{L_y}{S_{\text{start}} \cdot P_y} + \frac{L_y}{S_{\text{end}} \cdot P_y}\right) \right\rfloor$ which finally leads to

$$N_{\text{unit cell}} = N_{\text{unit cell}} \left\lfloor \frac{1}{2} \cdot \left(\frac{L_y}{S_{\text{start}} \cdot P_y} + \frac{L_y}{S_{\text{end}} \cdot P_y}\right) \right\rfloor (13).$$

For the investigated spectral gradient in **Figure 2** with $P_x = 2.4$ µm, $P_y = 4$ µm, $L_x = 600$ µm, $L_y = 150$ µm, $S_{\text{start}} = 1$, and $S_{\text{end}} = 1.1$, the number of unit cells along the x- and y-dimensions compute to $N_x = 238$, $N_{\text{unit cell y,avg}} = 35$, while the total number of unit cells is $N_{\text{unit cell}} = 8330$.

As discussed in the main text for spectral gradients in **Figure 2** the mode density per unit cell can be calculated via

$$\rho_{\mathrm{m}} = \frac{N_{\text{unit cell x}}}{N_{\text{unit cell}}} = \frac{1}{N_{\text{unit cell}}} = 2.9 \cdot 10^{-2} \ (14).$$

The coupling gradient presented in **Figure 3** has the dimensions $P_{\mathrm{x}} = 2.4$ µm, $P_{\mathrm{y}} = 4$ µm, $L_{\mathrm{x}} = 650$ µm, $L_{\mathrm{y}} = 150$ µm, the same number of unit cells $N_{\text{unit cell y}}$ in each column, and $\Delta S = 0$, so $S_{\text{start}} = S_{\text{end}} = 1$. ρ_{m} can be calculated, analog to the scaling gradient, via

$$\rho_{\mathrm{m}} = \frac{N_{\text{unit cell x}}}{N_{\text{unit cell}}} = \frac{1}{N_{\text{unit cell y}}} = 2.7 \cdot 10^{-2} \ (15).$$

For the dual gradient discussed in **Figure 4a-d**, each unit cell slightly differs from all other unit cells, resulting in an equal number of unit cells N_{total} and resonances within the gradient, thus

$$\rho_{\text{res}} = 1 \ (16).$$

In comparison, the monospectral gradient with $S = 1.05$ from **Figure 2** gives $\rho_{\mathrm{m}} = 4.7 \cdot 10^{-4}$. This means an improvement of the resonance density per unit cell ρ_{m} by a factor of 62, 57, and 2128 for the spectral, the coupling, and the dual gradient, respectively.

Note 6: Mode density comparison to literature

In our literature comparison, we focused on extended structures like metasurfaces and gratings to benchmark the mode density of our gradient metasurfaces, as individual resonators are challenging to compare. Although single resonators can exhibit a high mode density, when arranged into an array they need large interparticle distances to avoid scattering effects. This significantly lowers the overall density of resonators per unit area. The ideal interparticle distance varies and lacks a definitive standard, complicating the establishment of a reliable mode density metric that accounts for this factor. However, our subwavelength arrangement, where each resonator has a unique mode, suggests that our dual-gradient metasurfaces significantly surpass the mode density of arrays composed of individual, non-coupled resonators.

In **Table 1**, we present key parameters of the resonant nanostructures under consideration, including their Q-factor, estimated number of modes, and mode density. Calculating these values is challenging due to the variety of the systems analyzed. We attempted to estimate these parameters as accurately as possible, which involved treating 1D gratings as 2D metasurfaces with quadratic unit cells. This approach allowed us to calculate the effective total number of unit cells within the structure, providing a basis for our comparisons.

Note 7: Numerical and analytical study on concentration dependent sensitivity:

To clarify why the optimal far-field coupling (in our context, the asymmetry via θ) varies with the quantity of analyte for achieving maximum sensitivity, and to explain why a single θ value cannot universally yield the best sensitivity across different analyte concentrations, we plan to first examine this phenomenon numerically. This investigation will be followed by an analytical approach based on our TCMT model.

Numerical approach:

We simulated our dual ellipse metasurfaces with varying θ and PMMA coating thicknesses, mirroring our experimental conditions. Remarkably, the simulations reveal drastically different behaviors in sensing sensitivity (absorbance $A = -\log(R_C/R_0)$) between a lossless and lossy metasurface. For the lossy simulations, we set k_{Si} to the value of 0.03 estimated by comparison with the experiments (see **Figure S10**). **Figure S21a** shows A in relation to the PMMA thickness, for θ ranging from 5° up to 45°. Indeed, this scenario confirms the expectation that smaller θ values (and consequently higher Q-factors) enhance sensitivity across all PMMA thicknesses. However, upon introducing losses ($k = 0.03$) to the uncoated metasurface, we observe in **Figure S21b** a significant change: the θ value yielding optimal sensitivity varies with PMMA layer thickness, shifting from 7.5° to 25° as the layer thickness increases from 0.5 nm to 200 nm. Further, when finely resolving the angle θ for distinct PMMA thicknesses from 1 to 200 mm, clear maxima for A appear in **Figure S21c** (normalized to their maxima in d). This clearly supports our experimental findings of **Figure 5d** and e where the optimal pixel with the highest sensitivity within the gradient shift toward lower θ for increasing PMMA thicknesses. Thus, it illustrates the complex interplay between loss, asymmetry, and analyte quantity in determining sensing performance. Figure 5d presents the extracted optimal angle $\theta_{A,max}$ where A is maximal for various analyte layer thicknesses. If multiple layer thicknesses share the same $\theta_{A,max}$, only one binned data-point is shown at their corresponding average layer thickness.

Analytical approach:

We employ the TCMT framework detailed in **Supplementary Note 2**. Utilizing equation (6), for $\omega = \omega_0$ and $r_0 = 1$, the reflectance coefficient $r(\omega)$ is expressed by:

$$r = e^{i\varphi} + \frac{\gamma_{\mathrm{rad}}}{\gamma_{\mathrm{tot}}} = e^{i\varphi} + \frac{\gamma_{\mathrm{rad}}}{\gamma_{\mathrm{rad}} + \gamma_{\mathrm{para}}} \quad (17)$$

From this, we derive the reflectance R using equation (7):

$$R = |r|^2 = \left| \frac{\gamma_{\mathrm{rad}} + (\gamma_{\mathrm{rad}} + \gamma_{\mathrm{para}})e^{i\varphi}}{\gamma_{\mathrm{rad}} + \gamma_{\mathrm{para}}} \right|^2 \quad (18)$$

For sensing applications, we use the absorbance A, defined as the change in reflectance induced by the analyte:

$$A = -\log(R_C/R_0)$$

where R_0 and R_C differ only in their γ_{para} values, with R_0 using $\gamma_{\mathrm{para}} = \gamma_{\mathrm{int}}$ (γ_{scat} is set to zero for simplicity), and R_C using both $\gamma_{\mathrm{para}} = \gamma_{\mathrm{int}} + \gamma_{\mathrm{ana}}$. The loss induced by the analyte is represented by γ_{ana}. Therefore, the absorbance can be calculated as:

$$A = -\log(R_\mathrm{C}/R_0) = -\log \frac{\left|\frac{\gamma_\mathrm{rad} + (\gamma_\mathrm{rad} + \gamma_\mathrm{int} + \gamma_\mathrm{ana})e^{i\varphi}}{\gamma_\mathrm{rad} + \gamma_\mathrm{int} + \gamma_\mathrm{ana}}\right|^2}{\left|\frac{\gamma_\mathrm{rad} + (\gamma_\mathrm{rad} + \gamma_\mathrm{int})e^{i\varphi}}{\gamma_\mathrm{rad} + \gamma_\mathrm{int}}\right|^2} \quad (19)$$

To evaluate A as a function of γ_rad (asymmetry) for varying γ_ana (amount of analyte), we select appropriate values for γ_int from TCMT-fitted spectra with γ_rad values from 0 to 0.36, see **Figure S16**. A φ of 75° was found by repeating the TCMT fit with fixed φ for all asymmetries. For γ_ana representing loss induced by different analyte concentration values, we chose $\gamma_\mathrm{ana} = 0.0001 - 0.5$. **Figures S21a** to **S21d** show A for various γ_rad values, with **Figure S22a** as the ideal lossless case ($\gamma_\mathrm{int} = 0$) and **Figure S22b** to **d** with $\gamma_\mathrm{int} = 0.01$, 0.06, and 0.15 with **c** representing realistic losses ($\gamma_\mathrm{int} = 0.06$). Notably, the optimal A shifts with each fixed γ_ana, indicating a clear trend of increasing optimal γ_rad with increasing γ_ana. Further, increasing the initial losses of the system shifts the optimal γ_rad for all $\gamma_\mathrm{ana}$to higher values. These findings align well with our numerical results from **Figure S21**.

Combining simulation data with a direct TCMT analysis, we validate our experimental results, demonstrating that the optimal γ_rad for peak sensitivity is analyte-dependent. These results strengthen the significance of our dual-gradient metasurfaces for SEIRAS applications, as they ensure optimal sensitivity regardless of analyte concentration.

Note 8: Influence of the incident angle

As a first step we numerically investigated both TM and TE modes of an example monospectral BIC metasurface (S=1.01) for large and small angles of incidence (β), both plotted in **Figure S25a**. As expected, we observe considerable shifts of the BIC, especially for larger angles and TM polarized light, while for small angles and TE light, the resonance shifts only slightly, as evident from **Figure S25b**. This particularly indicates that for small angle spreads below two degrees, the BIC mode maintains a relatively constant spectral position, and the resonance is expected to maintain its amplitude and Q-factor.

As a next step, we investigate the angles of illumination within our measurement setup. It is important to mention that the Spero microscope is a brightfield reflectance microscope. Unlike techniques such as confocal microscopy, where focused illumination is key, our setup utilizes a defocused laser beam to ensure that the entire field of view (2x2 mm²) is illuminated homogeneously. Consequently, the angles of incidence and their spread at specific locations within the imaging plane is minimal. Since detailed information about the internal optical components of the Spero microscope is not public knowledge, we rely on the published data and our own measurements to estimate the upper limit for the incidence angles.

We measured the length of the object distance ($d_o = 45mm$) and utilizing the objective's magnification ($M = 4$) along with the lens formula $\frac{1}{f} = \frac{1}{d_o} + \frac{1}{d_i}$ and the magnification formula $M = \frac{d_i}{d_0}$ with d_i as the image distance, we can calculate the focal length to be 36 mm [5].

With the objective's NA of 0.15 corresponding to a maximal angle of incidence ($\beta_{\max}$) of 8.6°, trigonometry allows us to calculate the exit pupil radius with $r_\mathrm{p} = \tan \beta_{\max} \cdot f = 5.5mm$, see **Figure S26a**. While the actual diameter of the laser beam at the imaging plane is not specifically known, we can use the field of view to estimate a lower limit for the beam's diameter with a radius of $\sqrt{2}mm$. Given that the illumination is fairly uniform across the field of view, the real beam diameter is likely larger, resulting in an even narrower angular spread. This leads to an upper limit estimate for the effective maximal illumination angle $\beta_{\mathrm{eff,max}} = \tan^{-1} \frac{r_\mathrm{p} - r_\mathrm{FOV}}{d_o} =$ 5.2° and $x = \frac{d_o r_\mathrm{FOV}}{r_\mathrm{p} - r_\mathrm{FOV}} = 15.6mm$ as the offset of the imaging plane from the focal plane.

Assuming uniform laser illumination on the objective and geometric optics, the light rays are sketched in **Figure S26b**, with a maximum angle of 5.2°. It is apparent how the incidence angle varies with the in-plane position.

To calculate the actual angular spread in our measurements, we map the incidence angles across the entire 2x2mm^2 field, discretized by our 480x480 pixels in **Figure S27a** using small angle approximations. A smooth pattern with minimal angular variation between adjacent pixels is visible. In **Figure S27b** we show a zoomed in section corresponding to a 100x100 µm² field of view which is roughly the area over which we average the spectral response of the monospectral metasurfaces (150x150µm²) excluding 25 µm at each border to limit losses induced by the edges. Here the maximal illumination angle is just 0.25°. In **Figure S27c-e** we directly analyze to angle of incident light weighted by the number of pixels for the dual gradient (600x400 µm² of analyzed area, incidence angles up to 1.33°), the monospectral metasurface, and for four neighboring pixels, in c, d, and e, respectively. It is apparent that even within the dual gradient, the angular spread is minimal and within a range where we do not expect much broadening.

Next, we numerically investigate the effect the found angular spread has on the measured spectra. In **Figure S27f** and g TM and TE polarized light incident at 0° (gray) is compared with the unweighted averaged spectrum ($\theta = 20°$, $S = 1$, black) for an angular spread for a dual gradient metasurface (black). **Figure S27h** and **i** show the corresponding results for the monospectral metasurface. In **Figure S27e** a slight shift is visible which only insignificantly alters the linewidth or the resonance amplitude. For the other three cases, no change is visible.

Finally, we performed further experiments to investigate possible spectral shift caused by the illumination. For this purpose we measured the reflectance of the monospectral metasurfaces with S=1.01 of **Figure 2** at **5** different position within the field of view. The positions are indicated in **Figure S27a** with the five red squares. The central one in black, and 4 more, one at each corner of the field of view. **Figure S27j** shows the resulting reflectance spectra using the respective color code. While smaller spectral shifts are visible, with less than 15nm over the whole field of view which is much larger than even the dual gradient, this effect is neglectable, especially when considering the total spectral shifts within the dual gradient.

Supplementary Tables

Table 1: Comparison of the total number of resonances and the resonance density.

Resonator system	Resonance type	Spectral range	Exp. Q-factor	Number of modes	Mode density	Reference
**Individual spectral gradient (our work)**	BIC	Mid-IR	80	**238**	**$2.9 \cdot 10^{-2}$**	-
**Individual coupling gradient (our work)**	BIC	Mid-IR	170	**270**	**$2.7 \cdot 10^{-2}$**	-
**Dual gradient (our work)**	BIC	Mid-IR	170	**27,500**	**1**	-
Molecular sensor for SEIRA	BIC	Mid-IR	120	100	$9.1 \cdot 10^{-4}$	[6]
Pixelated RI sensor	BIC	Vis	180	12	$2.9 \cdot 10^{-6}$	[7]
Trapped Rainbow	Plasmonic grating	Vis	30	116	$1.9 \cdot 10^{-2}$	[8]
Multiple Surface lattice resonances	Plasmonic SLR	Near-IR	-	-	$1.7 \cdot 10^{-4}$	[9]
Plasmonic gradient	Plasmonic resonators	Vis	10	-	$2.5 \cdot 10^{-2}$	[10]
Dielectric spectral-gradient metasurface	BIC	Near-IR	75	1,476	$4.3 \cdot 10^{-3}$	[11]
Radial bound states in the continuum	BIC	Vis	500	-	$3.5 \cdot 10^{-2}$	[3]

Supplementary Videos

All videos were directly taken from the hyperspectral measurements, after a rotation by 20°, and cropping the displayed field of few.

Supplementary Video 1: Spectral gradient

Reflectance video of the three monospectral metasurfaces with scaling factors of 1.01, 1.05, and 1.09, and the spectral gradient of $S = 1.0 - 1.1$, shown in **Figure 2**. The reflectance color code spans values from 0 to 0.7, the start frame is at 5672 nm, and the end frame at 6569 nm.

Supplementary Video 2: Coupling gradient

Reflectance video of the coupling gradient (top) and the spectrally aligned coupling gradient (bottom), discussed in **Figure 3**. The reflectance color code spans values from 0 to 0.9, the start frame is at 5608 nm, and the end frame at 6319 nm.

Supplementary Video 3: Dual gradient

Reflectance video of the dual gradient discussed in **Figure 4**. The reflectance color code spans values from 0 to 0.7, the start frame is at 5574 nm, and the end frame at 7153 nm.

Supplementary Video 4: Dual gradient for molecular sensing, uncoated

Reflectance video of the uncoated dual gradient discussed in **Figure 5**. The reflectance color code spans values from 0 to 0.7, the start frame is at 5574 nm, and the end frame at 6588 nm.

Supplementary Video 5: Dual gradient for molecular sensing, coated

Reflectance video of the dual gradient discussed in **Figure 5,** coated with a 1% solution of PMMA. The reflectance color code spans values from 0 to 0.7, the start frame is at 5574 nm, and the end frame at 6588 nm.

Supplementary Figures

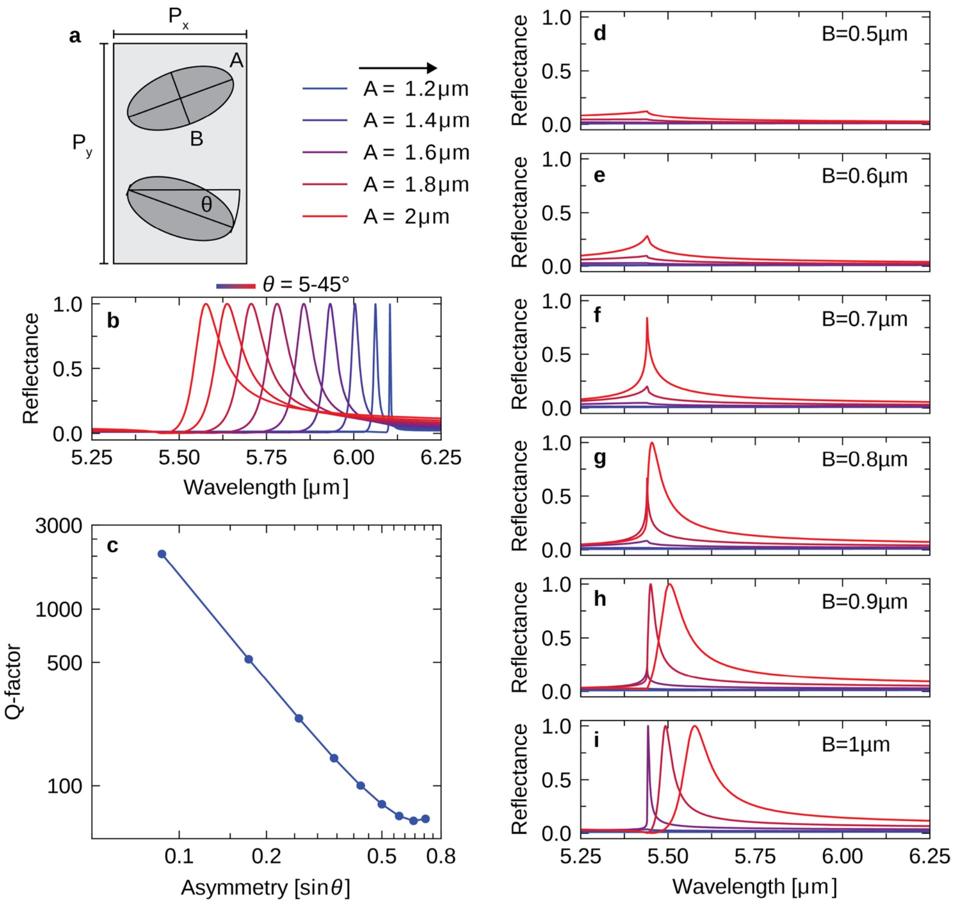

Figure S1: Numerical analysis of the dual-ellipse geometry. (a) Illustration of the tilted dual-ellipse unit cell, showing the short pitch P_x along the x-axis, and the long pitch P_y along the y-axis. The ellipse diameters of the long and short axes are labeled as A and B, respectively, with θ representing the tilt angle of the ellipse's long axis A relative to the x-axis. (b) Display of the tilt angle sweep, starting from $\theta = 5°$ (blue) to $\theta = 45°$ (red) in 5° increments. (c) Q-factors derived from (b) using TCMT fitting. Both axes are logarithmic, illustrating the typical symmetry-protected BIC relationship of $Q \sim \frac{1}{\alpha^2} \sim \frac{1}{sin^2(\theta)}$ as a linear plot with a slope of approximately -2. Deviations from this linear trend at large angles are attributed to the influence of the Rayleigh anomaly, as the BIC shifts towards it with increasing α. To achieve a wide range of Q-factors, we simulated 45° tilted ellipses of varying diameters A and B, while maintaining constant values for P_x, P_y, and the height. (d) – (i) Reflectance spectra for varying A from 1.2 to 2 µm in 5 steps and B from 0.5 to 1 µm in 6 steps. It's evident that combinations of small A and B do not exhibit a resonance, as they are blueshifted towards the Rayleigh anomaly, allowing energy to radiate away via the first diffraction order, preventing the formation of a strong BIC mode. Visible BIC resonances are only observed for larger A and B values. However, most visible modes have relatively high Q-factors due to their proximity to the Rayleigh anomaly[12]. The resonance for $A = 2$ µm and $B = 1$ µm is the only one with a sufficiently broad lineshape for our purposes. Larger structures were not considered to avoid

overlap at the tips of the ellipses at $\theta = 45°$. As shown in (c), the Q-factor deviation for high angles does not significantly stray from the ideal Q-factor – asymmetry relation.

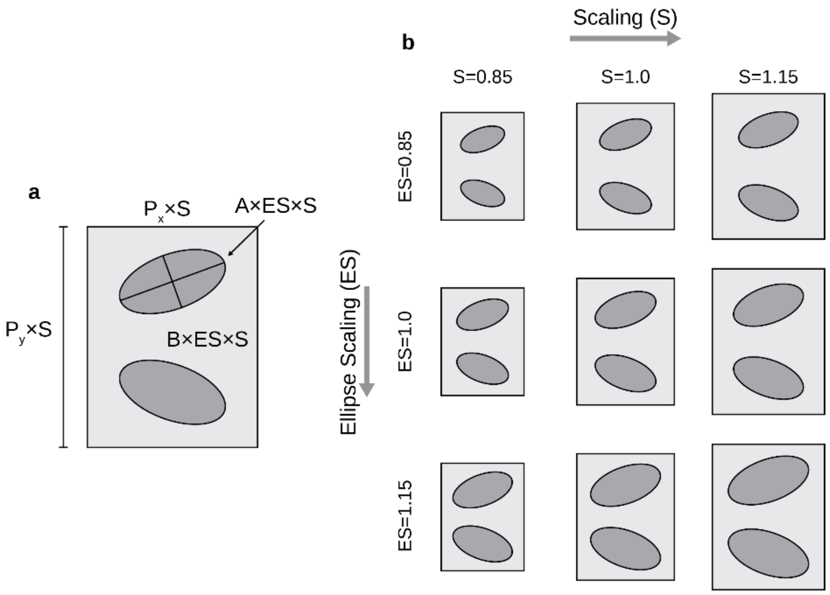

Figure S2: Illustration of the scaling factor S and the ellipse scaling factor ES. (a) sketch of the metasurface's unit cell, indicating the in-plane parameters along with the scaling factors affecting them. While S affects P_x, P_y, B, and A, ES alters A and B. (b) sketch showing the impact of the scaling factors on the geometrical parameters with arbitrary scaling factors of 0.85, 1, and 1.15 for S along the x-axis, and ES along the y-axis.

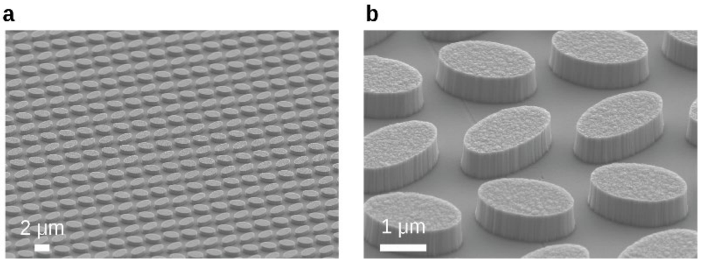

Figure S3: Spectral gradient SEM images. SEM images of the spectral gradient investigated in Figure 2 with $\Delta S = 0.1$, acquired at a tilting angle of 45°. Overview image in (a) and close-up in (b).

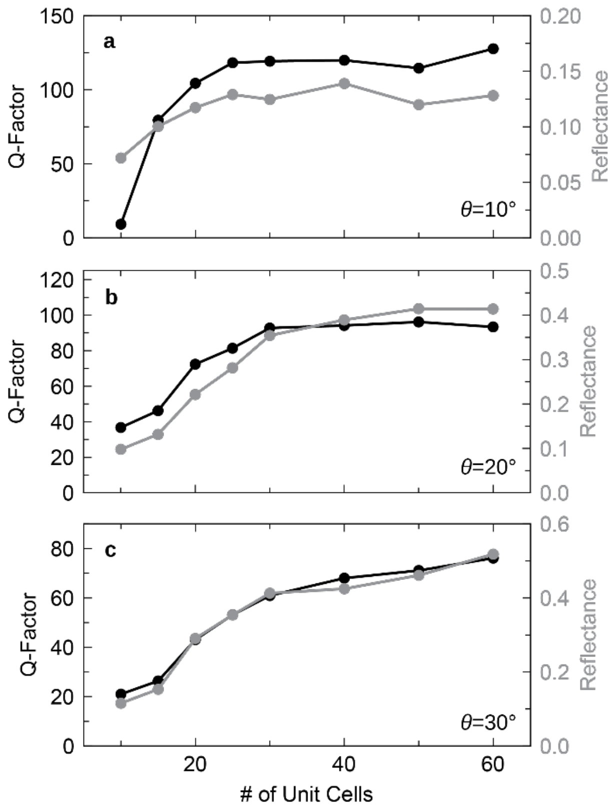

Figure S4: Monospectral metasurface size sweep for different asymmetries. Experimentally obtained and TCMT fitted Q-factors and reflectances for metasurfaces ranging from 10x10 to 60x60 unit cells for an ellipse tilting angle θ of 10, 20, and 30° in panel a, b, and c, respectively. An increase in both Q-factor and reflectance is evident as the array size grows. However, this increase becomes less pronounced for larger arrays. Specifically, for $\theta = 10°$, no noticeable improvement in either reflectance or Q-factor is observed beyond 25x25 unit cells. Conversely, for $\theta = 20°$, while reflectance continues to increase slightly for arrays larger than 30x30 unit cells, the Q-factor remains constant. Based on these findings, a size of 35 unit cells for the long axis dimension was selected for the monospectral metasurfaces. Note, we chose the same number of unit cells along the x- and the y-axis for simplicity. In fact the number of unit cells along the polarization direction is more crucial for the formation of the BIC mode[13].

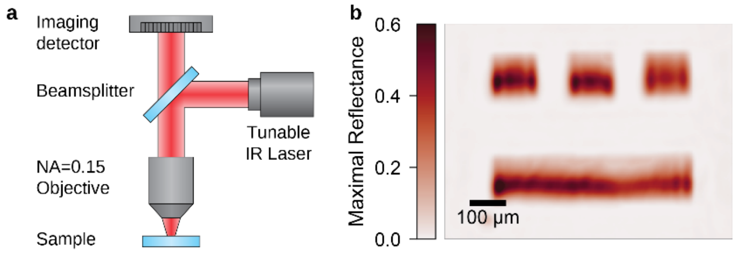

Figure S5: Optical characterization and reflectance map. (a) Sketch of the measurement setup. The light of three tunable quantum cascade lasers is guided via a dichroic mirror onto the sample through a 4x, $NA = 0.15$ objective. The reflected light is collected by the same objective and projected onto a 480x480 pixel detector. The lasers are adjusted in 2 cm^{-1} steps across the target spectral range, and an image is captured at each wavelength, resulting in a hyperspectral image. (b) Display of the maximum reflectance of each pixel of the spectral

gradient ($\Delta S = 0.1$) across wavelengths from 5.6 to 7 µm, illustrating consistently high reflectance amplitudes across the gradient.

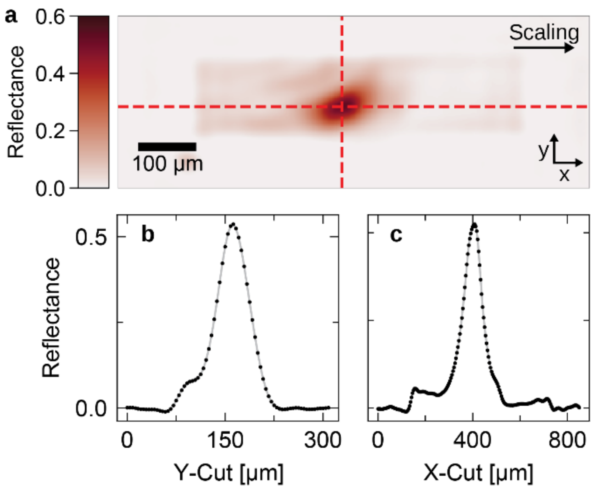

Figure S6: Cuts of the reflectance map of the spectral gradient. (a) Reflectance snapshot at 6.1 µm for the spectral gradient depicted in Figure 2 with $\Delta S = 0.1$. Specific cuts from the reflectance map in (a), corresponding to the two red dashed lines. (b) Vertical section along the y-axis, and (c) horizontal section along the x-axis.

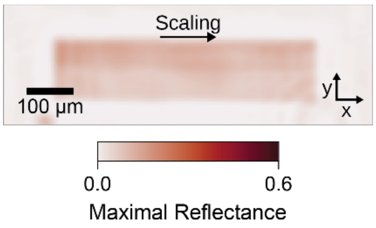

Figure S7: Effects of 90° rotated excitation polarization on a spectral gradient. Maximal reflectance maps of a spectral gradient with $\Delta S = 0.1$. The polarization of the electric field is rotated by 90° and is now parallel to the x-axis. Under this condition, no significant reflectance revealing a resonance is observed. The minor increase in reflectance within the gradient is attributed to the structured Si layer rather than the resonant effect.

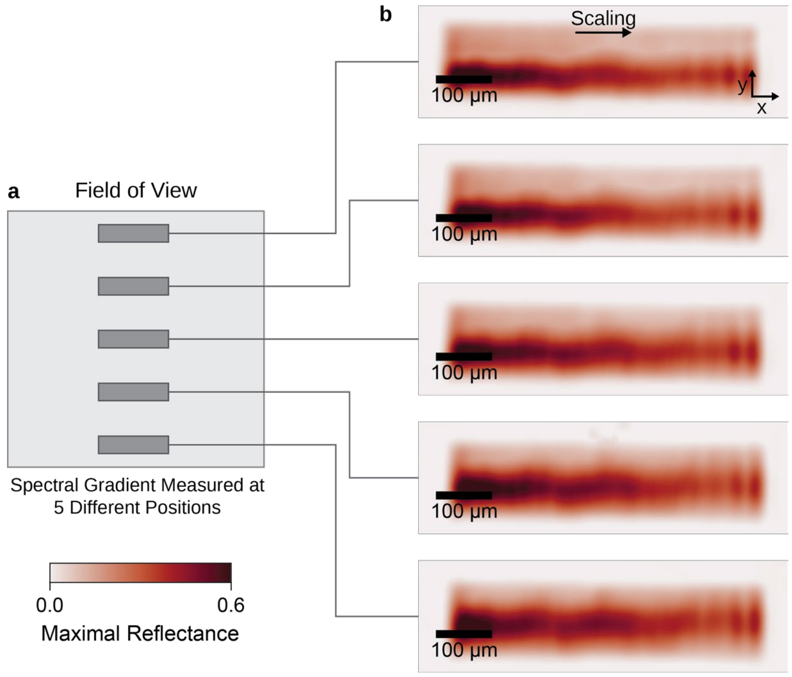

Figure S8: Reflectance patterns at different positions within the field of view. (a) Sketch of the five positions within the field of view where a spectral gradient with $S = 1 - 1.1$ was measured. These positions are central along the x-axis and spaced 350µm apart along the y-axis. (b) Maximal reflectance maps measured at these positions. While the overall amplitude remains constant across all positions, there is a noticeable shift in the area of maximal values along the y-axis within the gradients. For measurements taken at the lower part of the field of view, the highest values appear near the y-center of the gradient. As the measurement positions move upward, the highest values shift downward, approaching the bottom border of the gradient by the uppermost position. This effect is attributed to a slight tilt of the incident laser beam. Despite this shift, the maximal amplitude remains consistent across the field of view.

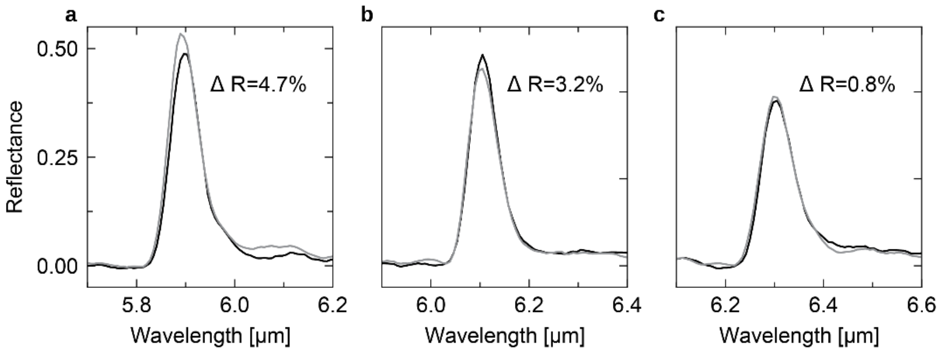

Figure S9: Reflectance comparison between monospectral and spectral gradient metasurfaces. Analogous to **Figure 2e**, this figure compares the spectral responses of monospectral metasurfaces with scaling factors S=1.01 (a), 1.05 (b), and 1.09 (c) in black against corresponding positions within the spectral gradient ranging from S=1.0 to 1.1 in gray. Unlike in **Figure 2e**, the reflectance amplitude is presented without normalization. The depicted difference in reflectance, marked in percentages within the plots, shows values below 5%. This minor variance, alongside nearly identical linewidths, showcases their almost identical farfield response. The slight difference in reflectance lies within the expected range of fabrication variation[14].

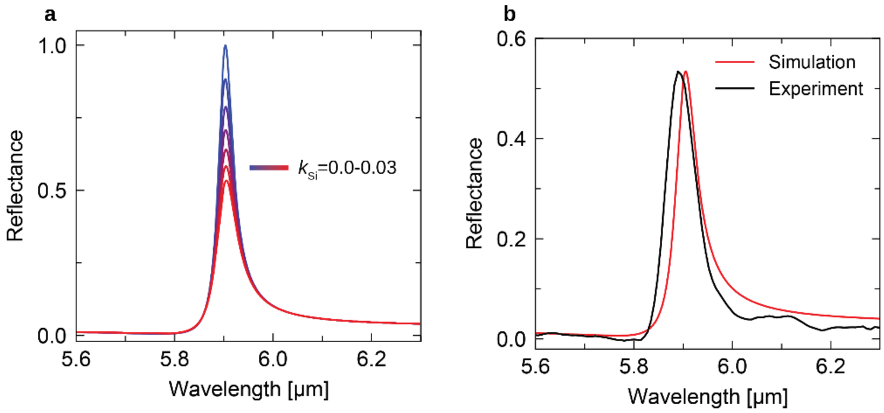

Figure S10: Comparison of simulations and experiments. (a) Reflectance spectra for simulated metasurfaces using $\theta = 20°$ and S=1.01. The imaginary part of the refractive index of Si k_{Si} is tuned from 0.0 to 0.03 in seven steps from blue to red, respectively. While the resonance position remains constant, the amplitude reduces from 1 to 0.53. (b) Direct comparison of the $k_{\mathrm{Si}} = 0.03$ simulated metasurfaces of (a) with the reflectance spectrum obtained in the spectral gradient of **Figure 2a-e** and **Figure S9a** with $\theta = 20°$ at the scaling position of $S = 1.01$. Good agreement is evident, even without additional adjustments of the numerical model.

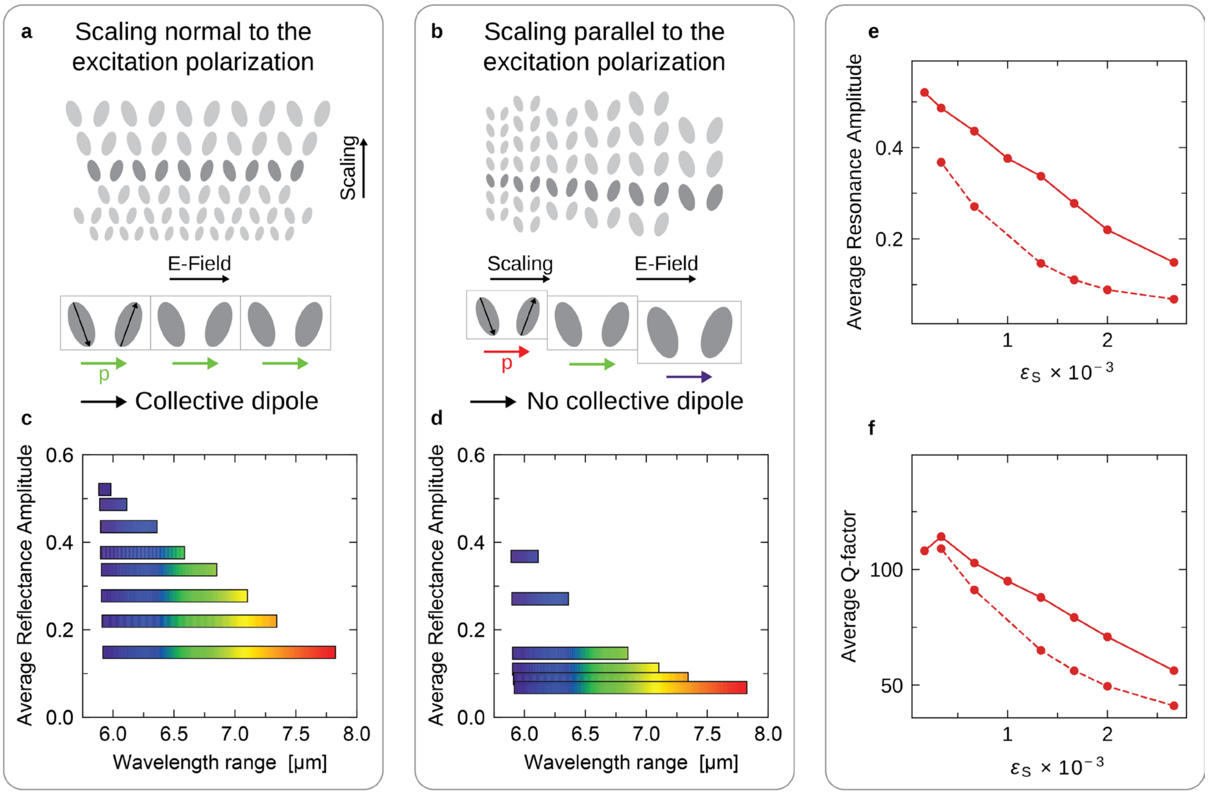

Figure S11: Scaling normal and parallel to the excitation direction. (a) Illustration of a spectral gradient with scaling perpendicular to the excitation polarization, allowing the formation of a collective dipolar mode across a chain of identical resonators. (b) Illustration of a spectral gradient with scaling parallel to the excitation polarization. This configuration hinders the collective dipole formation due to y-position mismatches in adjacent unit cells and their resonance wavelength offsets. (c) Graph showcasing the average reflectance amplitude and wavelength range for gradients with perpendicular scaling as in (a). Peak reflectance values correspond to the shortest wavelength range. (d) Average reflectance amplitude, analog to (c), but for gradients with parallel scaling as in (b). This configuration exhibits a notably reduced performance compared to the perpendicular scaling in (c). (e) Average resonance amplitude plotted against different ε_s for gradients with $\theta = 20°$. Solid lines represent perpendicular scaling to the excitation polarization, while dashed lines indicate parallel scaling. (f) Average Q-factors derived from TCMT fits for the spectral gradients, analog to (e).

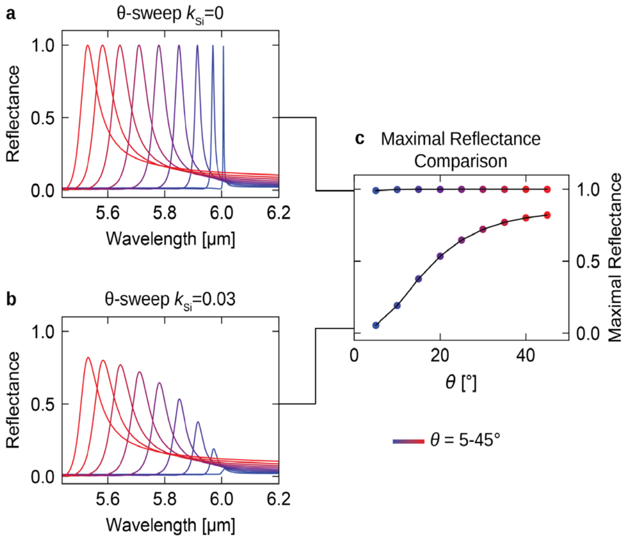

Figure S12: Influence of loss on BIC resonances with different asymmetries. Numerical analyses for $S = 1$ with varying asymmetry angle θ, ranging from 5° to 45° in increments of 5°, depicted from blue to red. (a) shows the scenario where the imaginary part of the refractive index of silicon is set to $k_{\mathrm{Si}} = 0$, demonstrating that all resonances achieve a reflectance amplitude of 1. In (b) the imaginary part of the refractive index is set to $k_{\mathrm{Si}} = 0.03$. It is apparent that the material losses dampen the resonances, leading to a reduced reflectance amplitude. Panel (c) compares the maximal reflectance between situation (a) and (b), highlighting the effect of material losses on reflectance and the steady increase of reflectance with θ for the lossy case, proving the increased susceptibility of high Q-factor resonances to loss.

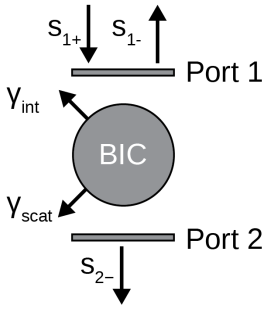

Figure S13: TCMT scheme. Schematic illustration of the used TCMT model consisting of a cavity which supports one resonance (BIC), two ports, one representing transmitted and one representing reflected light. Additionally, the cavity is connected to two parasitic loss channels, loss channels, the intrinsic loss γ_{int}, and scattering loss γ_{scat}.

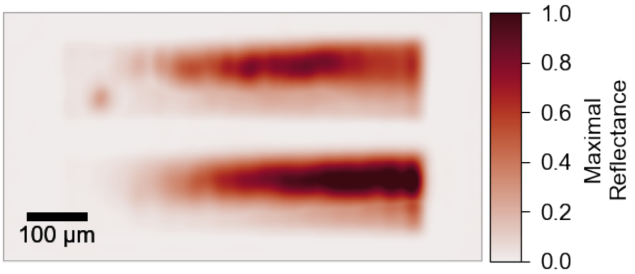

Figure S14: Maximal reflectance map of coupling gradient. Maximal reflectance value of each pixel of the two coupling gradients, presented in Figure 3. On the top the normal coupling gradient, and at the bottom, the spectrally-aligned coupling gradient.

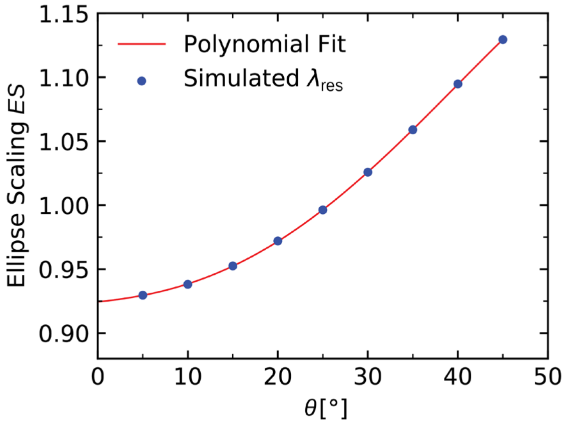

Figure S15: Dispersion correction for $\boldsymbol{\theta}$. Numerically retrieved ellipse scaling factors ES for periodic metasurfaces of varying ellipse tilting angle θ from 5 to 45° pictured as blue dots. The scaling factors were retrieved by finely sweeping ES for all θ to achieve resonances precisely at 5.8 µm. The numerical results were then fitted by a second order polynomial function, pictured as a red line, yielding: $y = 8.24 \cdot 10^{-5} \cdot x^2 + 9.99 \cdot 10^{-4} + 0.921$. These results were then used to fabricate the spectrally aligned coupling gradient in Figure 3f-k and the dual gradients in Figure 4.

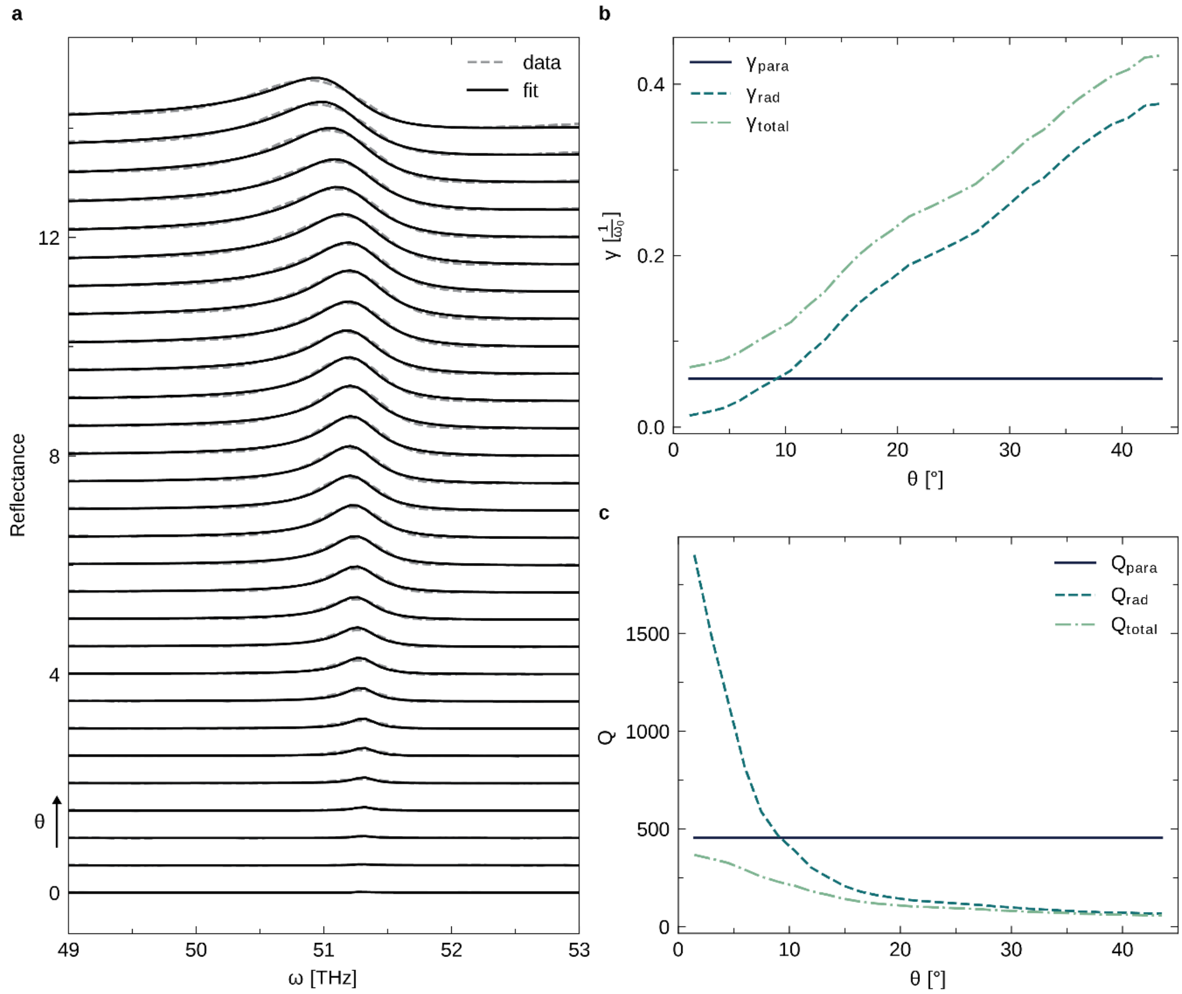

Figure S16: Study on parasitic losses in the coupling gradient. (a) Reflectance spectra of the spectrally-aligned coupling gradient of **Figure 3f-k** as gray dashed lines and the corresponding TCMT fits as solid black lines for a fixed γ_{para}. The spectra are taken along the x-axis of the gradient, with in total 29 steps (one step on each side of the gradient is excluded due to etch effects), each step roughly corresponding to a change of θ by 1.45°, the spectra are offset by 0.5 for clarity. (b) Extracted loss rates γ_{para} and γ_{rad}, and the combined loss rate γ_{total} plotted against the corresponding θ values. A clear crossing for γ_{para} and γ_{rad} is visible around $\theta =$ 9°. (c) Extracted Q-factors Q_{para}, Q_{rad}, and Q_{total}, analog to (b).

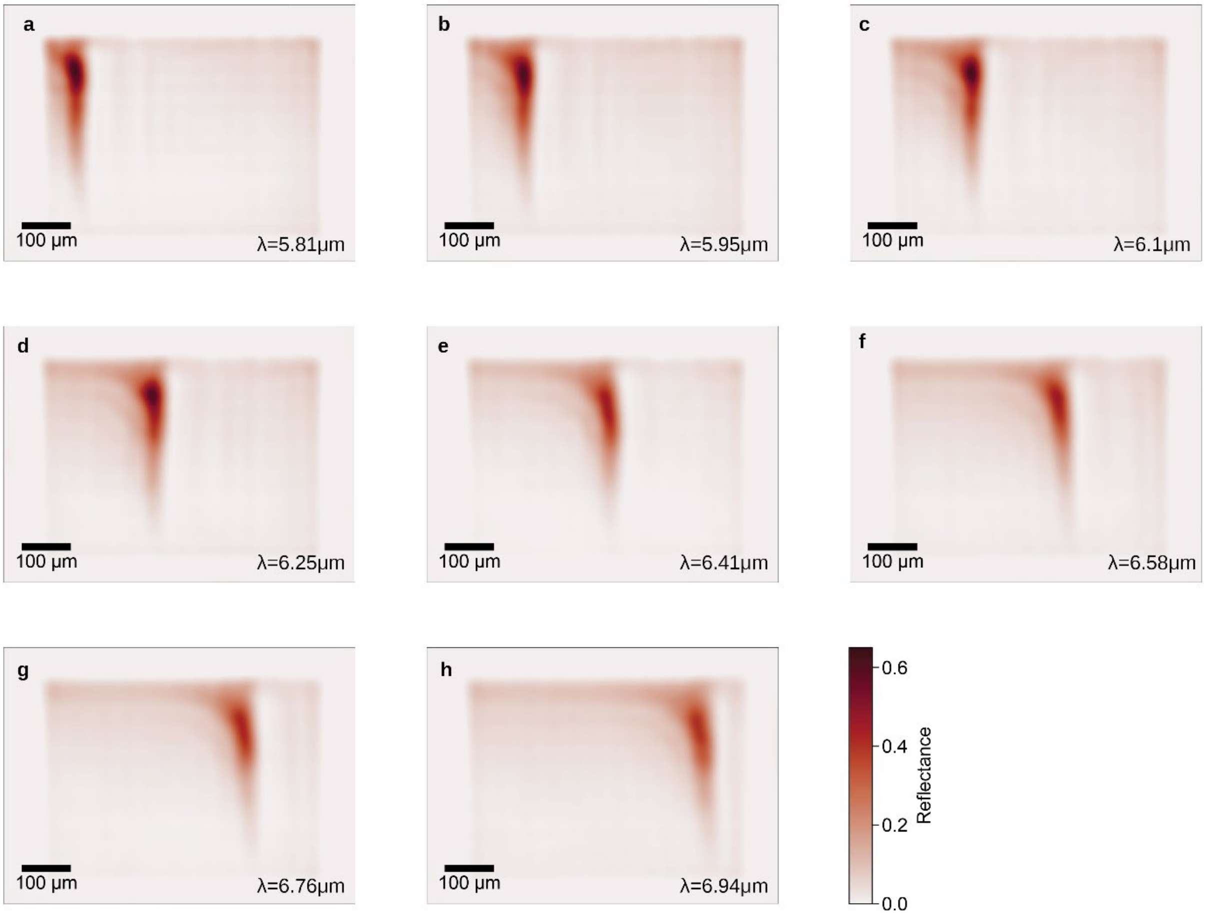

Figure S17: Single wavelength snapshots of the dual-gradient metasurface. Single wavelength snapshots of reflected light of the dual gradient discussed in **Figure 4a-d** with $S = 0.95 - 1.25$ and $\theta = 0 - 45°$ at a wavelength ranging from 5.81 in (a) to 6.94 µm in (h). Resonant sections within the gradient are apparent from high reflectance values.

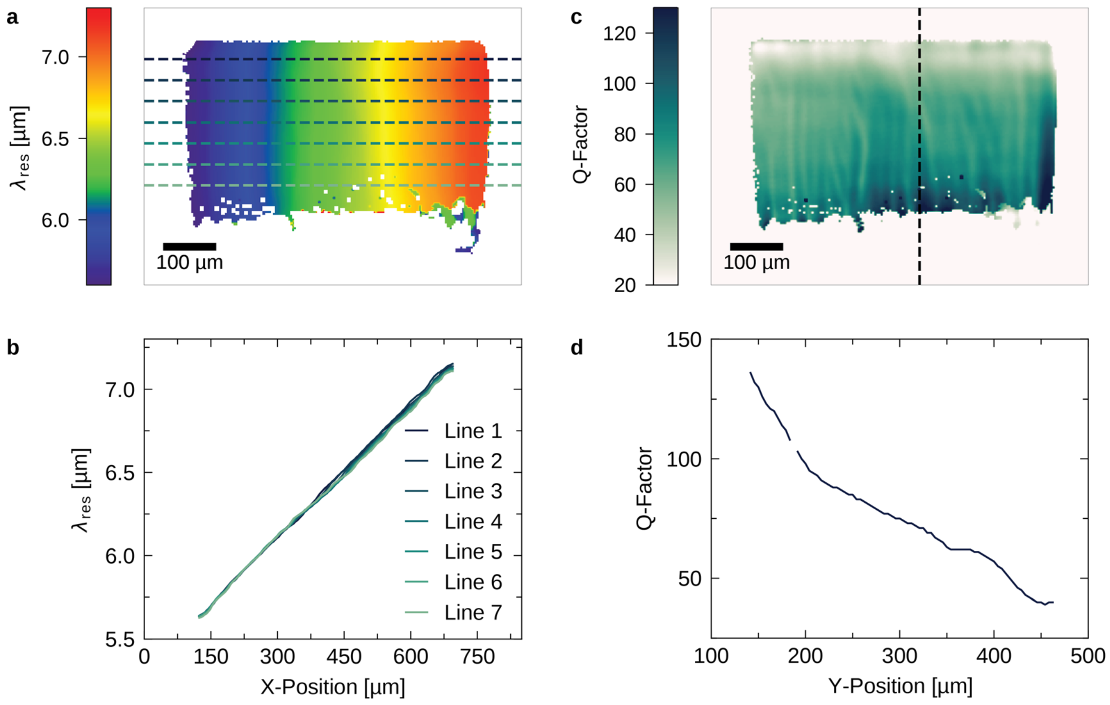

Figure S18: Resonance frequency and Q-factor slices of the $\Delta S = 0.3$ dual gradient. (a) Resonance frequency map of the dual gradient shown in **Figure 4a-d**. The seven horizontal dashed lines with a spacing of 10 pixels indicate the cuts taken along the scaling direction of the dual gradient. (b) Cuts of the map shown in (a) with the resonance wavelength plotted against the x-position. All seven plots show linear and uniform resonance wavelength shifts across the gradient, independent of the y-position within the gradient. (c) Q-factor map of the dual gradient shown in **Figure 4a-d**. (d) Q-factor cut along the y-axis of the map shown in (c) where the cut is indicated as a dashed line. A clear trend towards lower Q-factors for increasing asymmetry (increasing y-position) is visible. One point was removed from the cut, due to a fitting irregularity ($Q > 2000$).

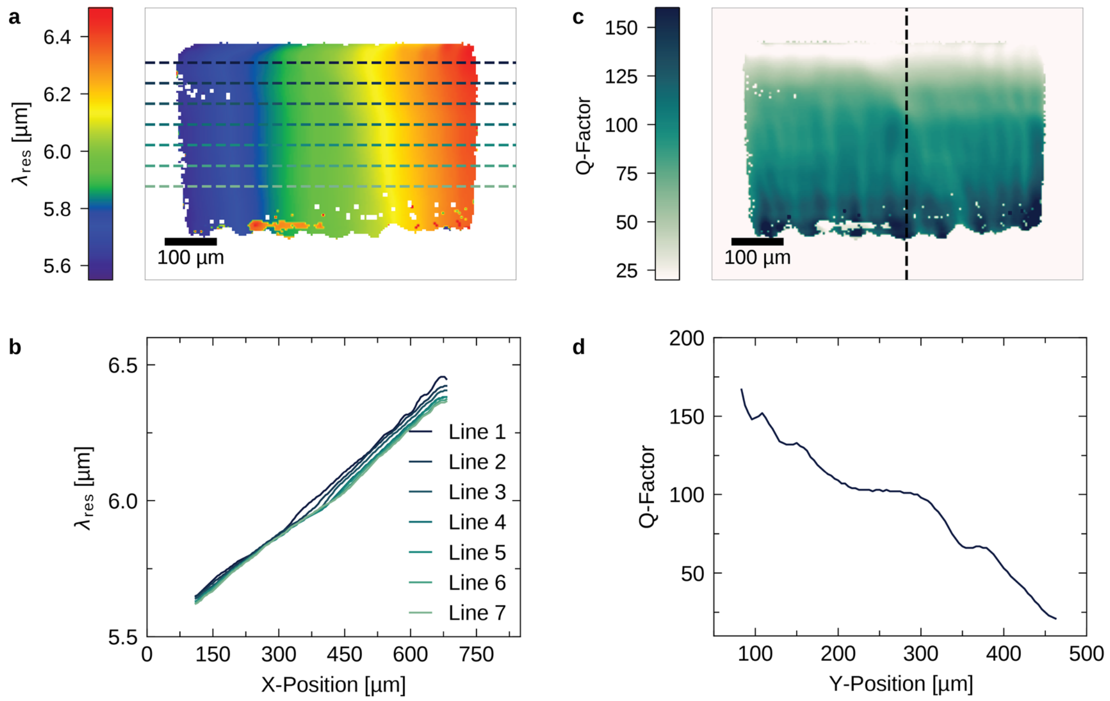

Figure S19: Cutting planes of the dual gradient for sensing. Resonance frequency and Q-factor slices of $\Delta S = 0.15$ dual gradient. (a) Resonance frequency map of the dual gradient shown in **Figure 4f-i**. The seven horizontal dashed lines with a spacing of 10 pixels, indicate the cuts taken along the scaling direction of the dual gradient. (b) Cuts of the map shown in (a) with the resonance wavelength plotted against the x-position. All seven plots show linear and uniform resonance wavelength shifts across the gradient, almost independent of the y-position within the gradient. (c) Q-factor map of the dual gradient shown in **Figure 4a-d**. (d) Q-factor cut along the y-axis of the map shown in (c) where the cut is indicated as a dashed line. A clear trend towards lower Q-factors for increasing asymmetry (increasing y-position) is visible.

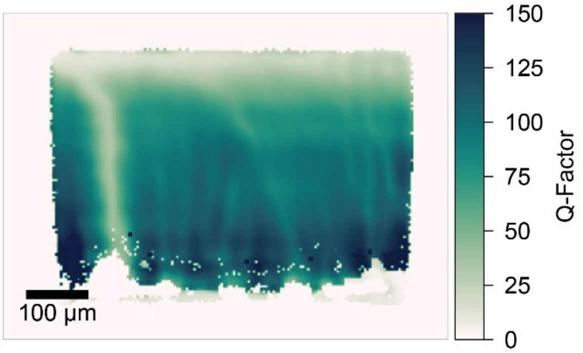

Figure S20: Q-factor map of coated dual gradient. Q-factor map of the dual-gradient metasurface of **Figure 4e-i** with an analyte concentration of 1%. The spectral fingerprint is clearly visible due to its strongly quenched Q-factors.

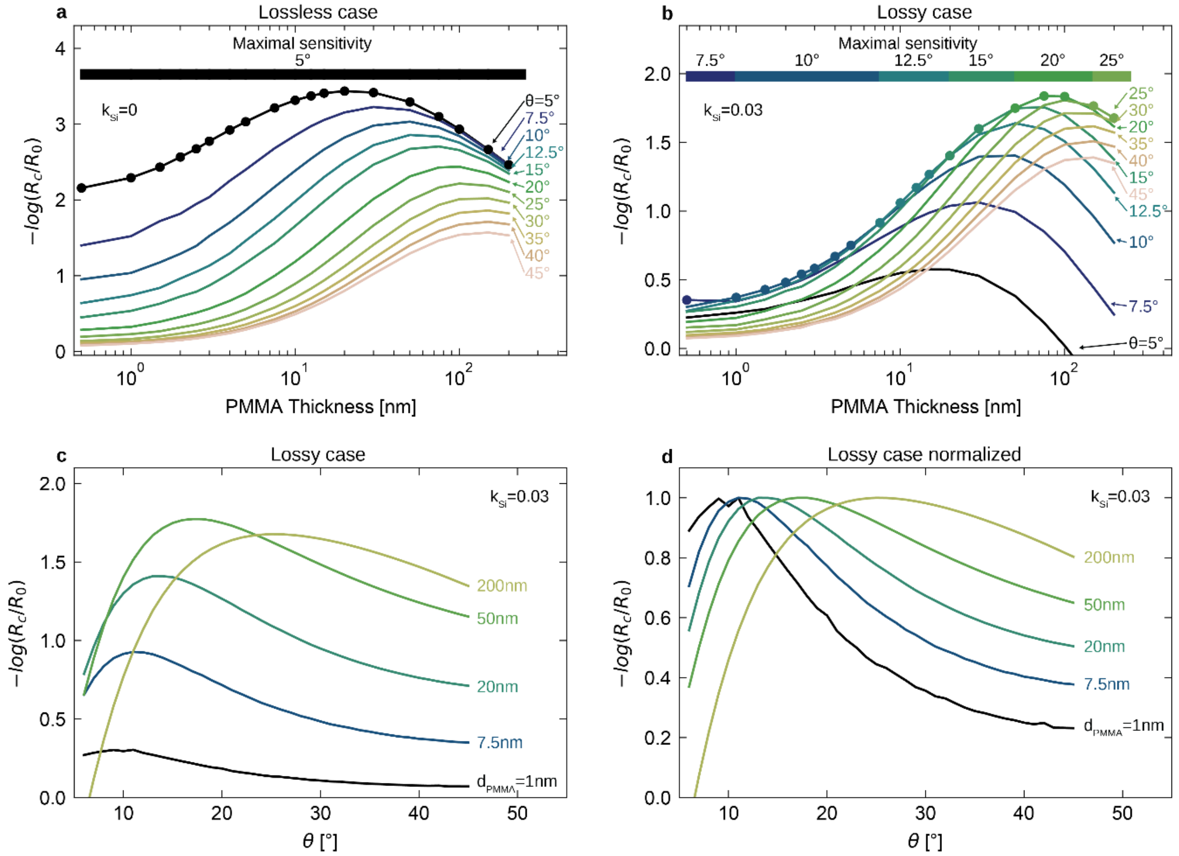

Figure S21: Numerical sensing study. Numerical absorbance results calculated using $A = -\log(R_C/R_0)$, where R_C and R_0 represent the reflectance amplitude at the resonance position for the coated and uncoated cases, respectively. (a) displays results for a lossless metasurface ($k_{Si} = 0$), plotting absorbance A as a function of PMMA coating thickness (ranging from 0.5 nm to 200 nm) across various angles θ from 5° to 45° in 5° increments. Each line, corresponding to a specific θ, is marked with dots at points indicating the highest performance (highest A) for a given PMMA thicknesses, with the optimal angle for each thickness region highlighted at the top of the graph. An increase in absorbance with PMMA thickness up to layers above 100 nm is observed, with the metasurface at $\theta = 5°$ showing superior sensitivity across all layer thicknesses, suggesting that higher Q-factors enhance sensitivity in a lossless metasurface

scenario. Panel (b) introduces silicon loss ($k_{\mathrm{Si}} = 0.03$) to better align with experimental conditions, see **Figure S10**. In this context, there is no single θ that consistently outperforms across the various PMMA thicknesses, but the optimal angle rather shifts between 7.5° and 25° for the thicknesses simulated, as already expected from experiments. A detailed analysis for selected PMMA layers (1, 7.5, 20, 50, and 200 nm) is shown in (c) with a higher θ resolution in 1° steps. Absorbance A is normalized to the maximum A for each layer thickness to allow comparison in (d). Notably, a distinct optimal θ is apparent for each PMMA layer thickness, validating experimental observations in **Figures 4d** and **4e**, where optimal sensing performance shifts to regions of higher θ and lower Q-factors for increasing PMMA thicknesses.

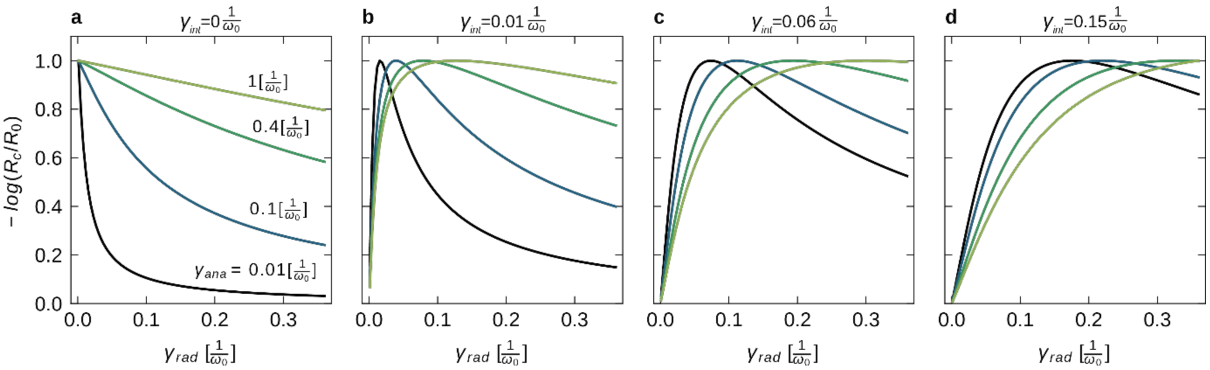

Figure S22: Analytical sensing study. Dependence of absorbance A on the radiative decay rate γ_{rad} for various analyte-induced loss rates γ_{ana} (from 0.01 to 1). γ_{rad} ranging from 0 to 0.35 is based on experimental findings presented in **Figure S16**. Panel (a) shows the normalized absorbance for the idealized lossless scenario ($\gamma_{\mathrm{int}} = 0$), demonstrating that the highest sensitivity across all γ_{ana} values is observed at the lowest γ_{rad}, with a rapid decline in sensitivity as γ_{rad} increases. Panels (b) to (d) show the normalized absorbance with γ_{int} = 0.01, 0.06, 0.15, revealing a complex relationship where each γ_{ana} achieves maximum sensitivity at a distinct γ_{rad}. The optimal γ_{rad} is shifting to higher values when γ_{int} increases, showcasing the transformation from the lossless case in (a) to realistic losses in (c) (following **Figure S16)** and beyond.

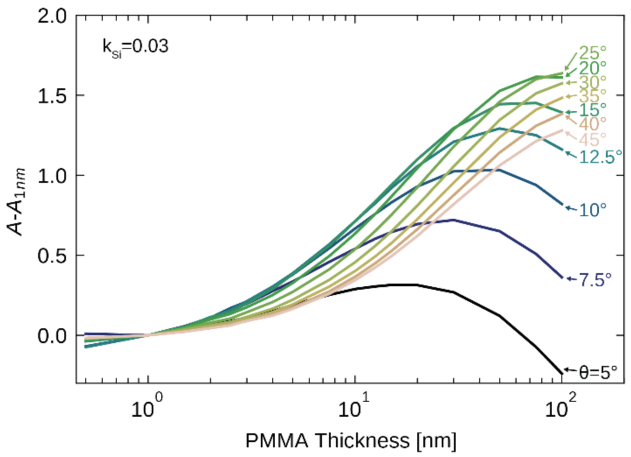

Figure S23: Calibration Curve. Calibration Curve based on the numerical results of **Figure S21b** for various asymmetries (θ), for PMMA thicknesses from 0.5nm to 100nm. ΔA is defined as $\Delta A = A - A_{1nm}$ using the absorbance of a 1 nm PMMA film A_{1nm} as reference absorbance. An unambiguous relationship between concentration and optical signal is visible.

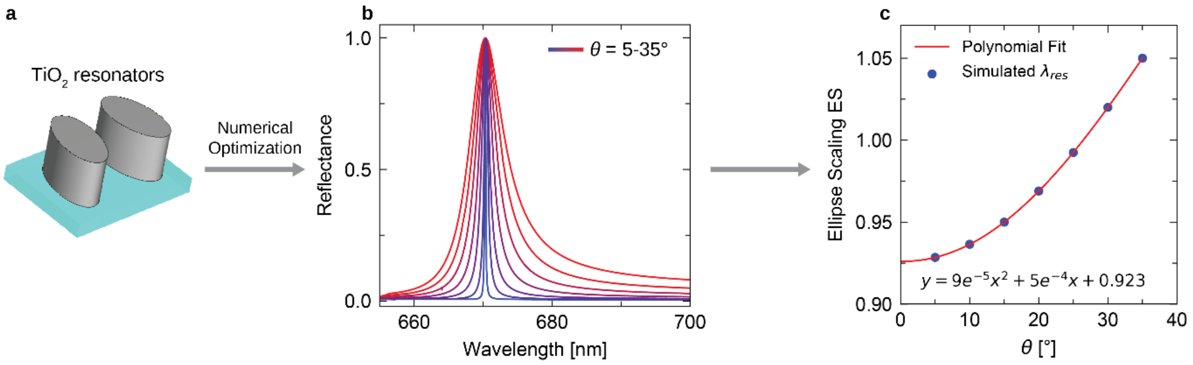

Figure S24: Simulation study of visible BIC geometries. Tilted ellipse geometry sketched in (a). For the simulation TiO_2 with $n = 2.54$, $k = 0$, and SiO_2 as the substrate with $n = 1.46$, $k = 0$ was used. The structural parameters, as detailed in **Figure S1a**, are set to $P_x = 330nm$, $P_y = 450nm$, A=260nm, B=140nm, and $h = 160nm$. The scaling factor ES, adjusting the two diameters of the ellipse, was varied from 0.9285 ($\theta = 5°$) to 1.05 ($\theta = 35°$) to counteract the dispersion induced by the asymmetry sweep. The resulting dispersion corrected reflectance spectra for $\theta = 5 - 35°$ are displayed in (b), where pronounced reflectance peaks with negligible resonance shifts across different asymmetries demonstrate that the gradient principle can be applied to visible wavelengths. (c) shows the second-order polynomial fit, which enables the construction of a dual gradient around 670 nm.

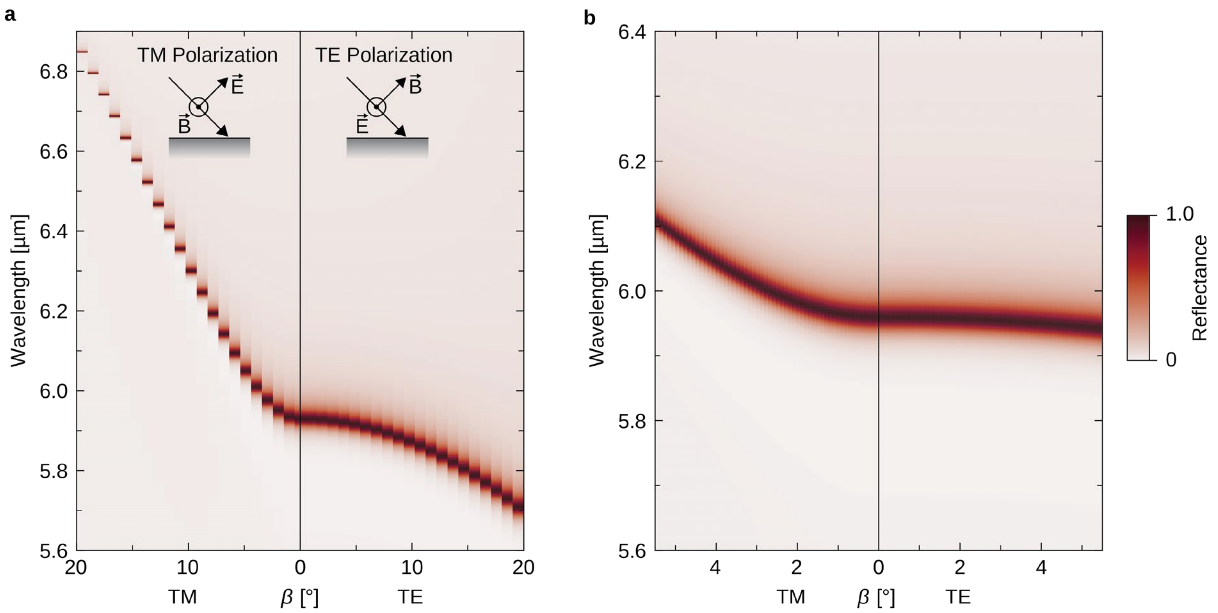

Figure S25: Incidence angle sweeps. Numerical incidence angle β sweeps with $\theta = 20°$ and $S = 1$ for both TM (in-plane k-vector) and TE (no in-plane k-vector) polarized light. In (a) sketch of TM and TE polarization at the top and a wide spectral reflectance sweep of 20°, with a strongly redshifting BIC for TM light, while the TE polarized light shift less and to short wavelengths. In (b) a zoomed in section of (a) up to 5.5°. Here the BIC mode for TM polarized light maintains its spectral position for angles below 1°, and for TE polarized light, the position remains constant throughout the range up to 5.5°.

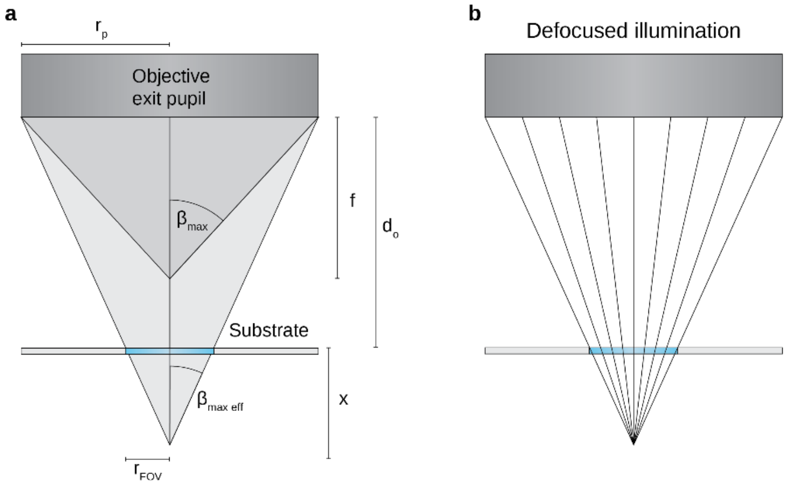

Figure S26: Focusing conditions of the microscope. (a) sketch of the microscope's focusing configuration. The maximal angle of incidence $\beta_{\max}$ is set by the 0.15 NA of the objective. The laser beam illuminating the sample is defocused as illustrated with the light gray cone. For a lower limit estimate, we set the beam diameter at the substrate to the position of the outermost pixel within our field of view, which results in a beam radius of $\sqrt{2}$ mm. (c) illustrates the geometric beam paths for defocused illumination.

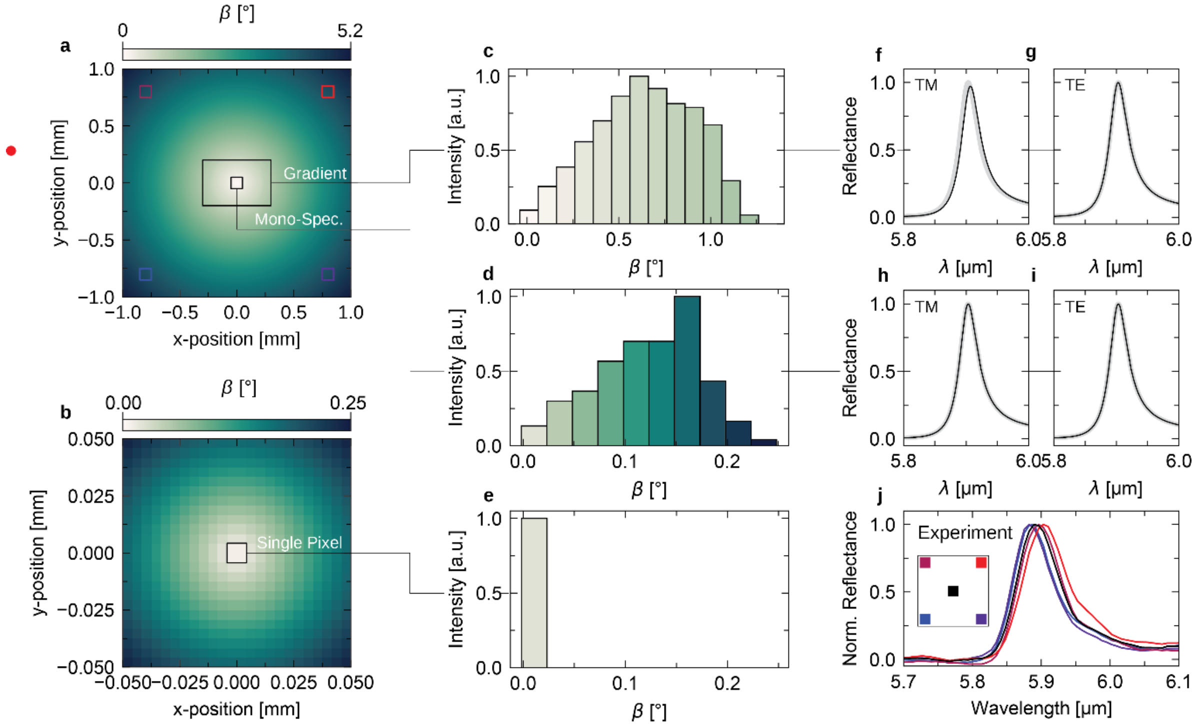

Figure S27: Angular distribution of incoming light intensity. The angular distribution of incoming light intensity under plane wave illumination, geometric optics and small angle approximation, considering the objective configuration depicted in **Figure S26** with a maximal angle of 5.2° at a $\sqrt{2}$ mm distance from the origin (edge of the field of view). (a) distribution across a 2x2 mm^2 area, equivalent to the microscope's field of view, divided into 480x480 pixels to represent the camera's resolution, with each pixel approximately 4.3 μm^2 in size. The angular transition across this field is smooth, with minimal variation of angles within small sections such as the marked areas of the dual gradient and monospectral metasurface of **Figure 2**, **4**, and **5**, excluding a border of 25 µm on all sides. (b) Close up of the central 100x100 μm^2 area, representing the dimensions of the monospectral metasurfaces, where the maximal incidence angle reaches just 0.25°. (c) Angular distribution for the dual gradient area marked in (a), with a total angle spread visible between 0.05 to 1.33°. In contrast, section (d) shows the monospectral gradient, revealing an angle spread from 0.025 to 0.25°. Section (e) zoomes in on 4 neighboring pixel equivalent to the rectangle in (b). The minimal angles vary slightly across (c) to (e) due to different histogram binning. Sections (f) and (g) compare TM and TE polarized light, respectively, normalized over the angle range from 0 to 1.33° to match the dual gradient's angular spread shown in black, against the reflectance at 0° incidence in gray. While TM polarization shows a visible but minimal deviation, TE polarization shows no change. (h) and (i) extend this analysis to the monospectral metasurface, showing no deviation from angled to 0° incidence for both TM and TE polarization.

Supplementary References

1. Fan, S., Suh, W. & Joannopoulos, J. D. Temporal coupled-mode theory for the Fano resonance in optical resonators. *J. Opt. Soc. Am. A* **20**, 569 (2003).

2. Suh, W., Wang, Z. & Fan, S. Temporal coupled-mode theory and the presence of non-orthogonal modes in lossless multimode cavities. *IEEE J. Quantum Electron.* **40**, 1511–1518 (2004).

3. Kühner, L. *et al.* Radial bound states in the continuum for polarization-invariant nanophotonics. *Nat. Commun.* **13**, 4992 (2022).

4. Sidorenko, M. S. *et al.* Observation of an Accidental Bound State in the Continuum in a Chain of Dielectric Disks. *Phys. Rev. Appl.* **15**, 1–11 (2021).

5. Hecht, E. *Optik*. (De Gruyter, 2018). doi:10.1515/9783110526653.

6. Tittl, A. *et al.* Imaging-based molecular barcoding with pixelated dielectric metasurfaces. *Science (80-.).* **360**, 1105–1109 (2018).

7. Jahani, Y. *et al.* Imaging-based spectrometer-less optofluidic biosensors based on dielectric metasurfaces for detecting extracellular vesicles. *Nat. Commun.* **12**, 4–13 (2021).

8. Zhou, L. *et al.* Super-Resolution Displacement Spectroscopic Sensing over a Surface "Rainbow". *Engineering* **17**, 75–81 (2022).

9. Reshef, O. *et al.* Multiresonant High- Q Plasmonic Metasurfaces. *Nano Lett.* **19**, 6429–6434 (2019).

10. Xu, T., Wu, Y. K., Luo, X. & Guo, L. J. Plasmonic nanoresonators for high-resolution colour filtering and spectral imaging. *Nat. Commun.* **1**, 59 (2010).

11. Jangid, P. *et al.* Spectral Tuning of High-Harmonic Generation with Resonance-Gradient Metasurfaces. *Adv. Mater.* (2023) doi:10.1002/adma.202307494.

12. Zheng, P. *et al.* Plexcitonic Quasi-Bound States in the Continuum. *Small* **17**, (2021).

13. Gölz, T. *et al.* Revealing mode formation in quasi-bound states in the continuum metasurfaces via near-field optical microscopy. 1–30 (2024) doi:10.48550/arXiv.2404.17346.

14. Kühne, J. *et al.* Fabrication robustness in BIC metasurfaces. *Nanophotonics* **10**, 4305–4312 (2021).
